# Functional and Structural Characterization of Clinical-Stage Janus Kinase 2 Inhibitors Identifies Determinants for Drug Selectivity

**DOI:** 10.1021/acs.jmedchem.4c00197

**Published:** 2024-06-06

**Authors:** Ya Miao, Anniina Virtanen, Jakub Zmajkovic, Morgane Hilpert, Radek C. Skoda, Olli Silvennoinen, Teemu Haikarainen

**Affiliations:** † Faculty of Medicine and Health Technology, Tampere University, 33520 Tampere, Finland; ‡ Institute of Biotechnology, HiLIFE, University of Helsinki, 00790 Helsinki, Finland; § Experimental Hematology, Department of Biomedicine, University Hospital Basel and University of Basel, 4056 Basel, Switzerland; ∥ Fimlab Laboratories, 33520 Tampere, Finland

## Abstract

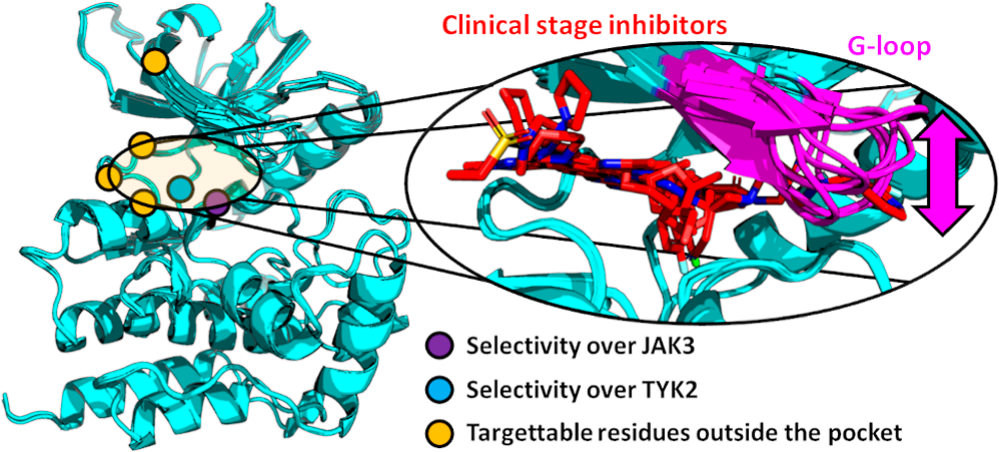

Janus kinase 2 (JAK2) plays a critical role in orchestrating hematopoiesis, and its deregulation leads to various blood disorders, most importantly myeloproliferative neoplasms (MPNs). Ruxolitinib, fedratinib, momelotinib, and pacritinib are FDA-/EMA-approved JAK inhibitors effective in relieving symptoms in MPN patients but show variable clinical profiles due to poor JAK selectivity. The development of next-generation JAK2 inhibitors is hampered by the lack of comparative functional analysis and knowledge of the molecular basis of their selectivity. Here, we provide mechanistic profiling of the four approved and six clinical-stage JAK2 inhibitors and connect selectivity data with high-resolution structural and thermodynamic analyses. All of the JAK inhibitors potently inhibited JAK2 activity. Inhibitors differed in their JAK isoform selectivity and potency for erythropoietin signaling, but their general cytokine inhibition signatures in blood cells were comparable. Structural data indicate that high potency and moderate JAK2 selectivity can be obtained by targeting the front pocket of the adenosine 5′-triphosphate-binding site.

## Introduction

Janus kinase (JAK) family of nonreceptor tyrosine kinases consists of JAK1–3 and TYK2 (tyrosine kinase 2) that serve as triggering kinases for cellular signaling of over 50 cytokines and hormones in regulation of blood and immune cells and metabolism. JAK2 activity is crucial for hematopoietic signaling particularly in homomeric myeloid, and hormone receptors and activating mutations of JAK2 have been identified in multiple malignancies, most notably Philadelphia chromosome-negative myeloproliferative neoplasms (MPNs), where mutated JAK2 drives the disease manifestations.^1−3^ MPNs are clonal neoplastic diseases initiated in the bone marrow from a mutated hematopoietic stem cell.^4^ MPNs are classified into three subtypes: polycythemia vera (PV) and essential thrombocythemia (ET), both with a prevalence of ∼160,000 cases in the USA, and primary myelofibrosis (PMF) with a prevalence of ∼16,000 cases.^5^ The activating JAK2-V617F mutation is responsible for over 95% of PV and more than 50% of the ET and PMF cases. Other pathogenic MPN mutations in calreticulin (CALR) and thrombopoietin receptor (MPL) genes also cause MPN by activating JAK2 signaling.^6,7^ In addition, mutations in JAK2 have been linked to other hematologic malignancies, such as acute lymphoblastic leukemia, acute myeloid leukemia, and acute megakaryoblastic leukemia.^8−10^

**Figure 1:**
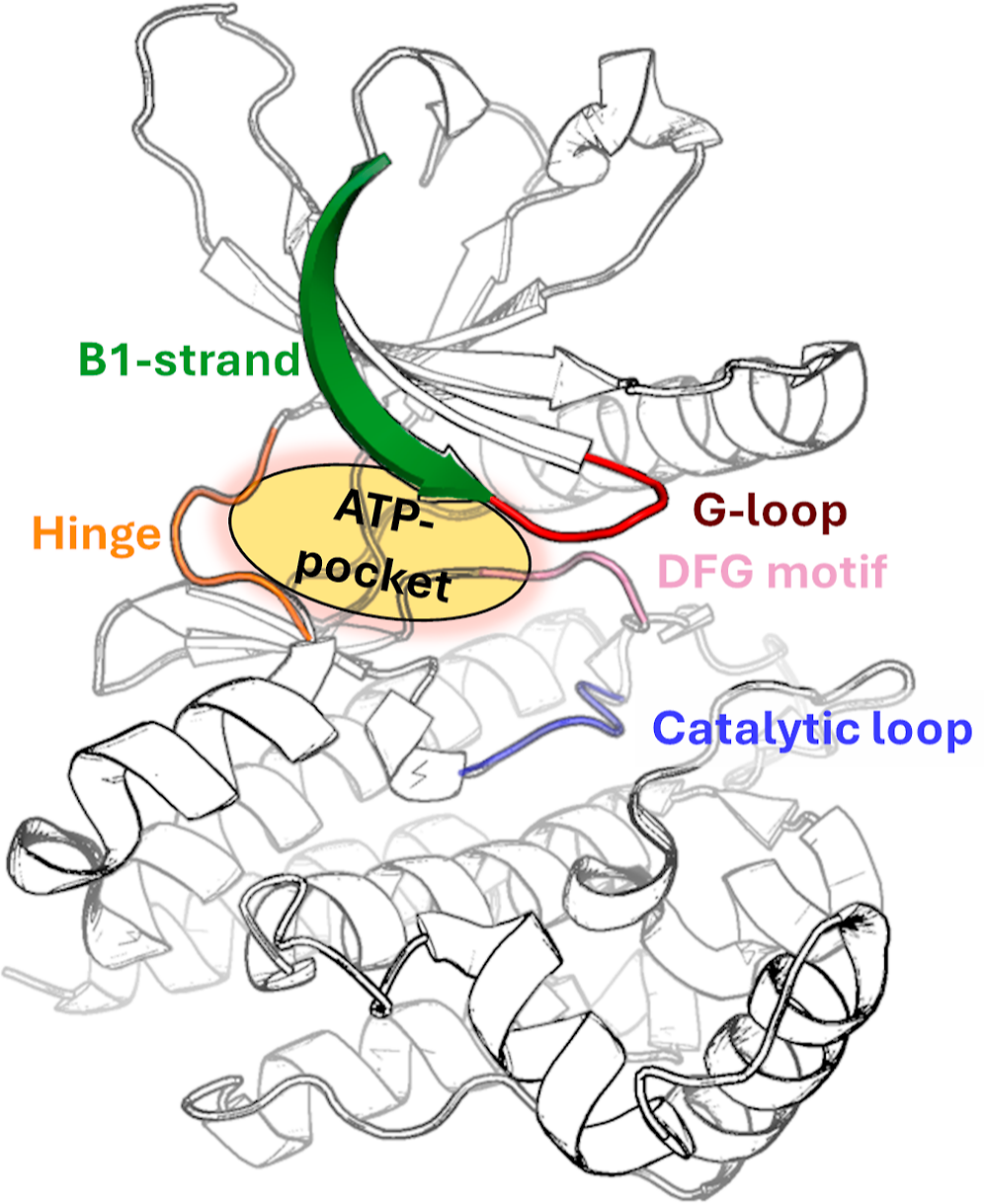
Structure of the JAK2 kinase domain. ATP pocket and close-by structural motifs are highlighted.

JAK2 has become an important therapeutic target and four JAK2 inhibitors, ruxolitinib, fedratinib, pacritinib, and momelotinib, have been approved for the treatment of MPNs, and several other compounds are in late-stage clinical development.^11^ All of these compounds are type-I inhibitors that target the conserved active conformation of the adenosine 5′-triphosphate (ATP)-binding pocket of the kinase domain and show variable target selectivity and differences in clinical profiles. ATP pocket is a key druggable target in kinases (Figure 1). It is lined by a hinge region, a β1-strand, a flexible G-loop, a catalytic loop, and a conserved DFG-motif within the activation loop. Although the pocket is well-suited for binding small-molecule drugs, its high conservation in the kinome makes the development of highly selective inhibitors challenging.

Upon approval in 2011, the JAK1/JAK2 inhibitor ruxolitinib revolutionized the treatment of myelofibrosis as the first targeted therapeutic for the disease and is currently approved for primary/secondary myelofibrosis (MF) and hydroxyurea-resistant PV as well as for applications in graft-versus-host disease (GVHD).^12^ Fedratinib was approved by the FDA in 2019 and is effective both as a first-line therapy in JAK inhibitor-naïve patients and as a second-line therapy for MF patients intolerant or resistant to ruxolitinib.^13,14^ Pacritinib is also a treatment option for MF patients with thrombocytopenia.^15−17^ Many MF patients suffer from anemia, and momelotinib is an emerging drug for these patients. By inhibiting the activin receptor type-1 (ACVR1) and reducing hepcidin, momelotinib increases iron availability and thereby ameliorates anemia.^18^ There are also several JAK2 inhibitors (JAKinibs) that are or have been in late-stage clinical trials for hematological diseases (MPN, leukemia, multiple myeloma, and lymphoma): lestaurtinib, itacitinib, ilginatinib, gandotinib, cerdulatinib, and AT9283. Despite the efficacy of the MPN-approved or -evaluated JAKinibs in relieving symptoms and improving blood counts, the JAK2 selectivity of inhibitors, if any, is relative rather than absolute and JAK1-mediated off-target effects such as immune suppression with impaired antimicrobial responses are common.^19,20^

The development of next-generation JAK2 inhibitors requires information on potency, selectivity, and binding modes of the existing inhibitors. To this end, we performed a comprehensive analysis of 10 JAK inhibitors currently in clinical use or in late-stage clinical trials by profiling in vitro their JAK isoform, cytokine signaling, and JAK2 V617F selectivity. To gain insights into the binding mechanism and selectivity of the inhibitors, we determined the crystal structures of seven clinical-stage JAK2 inhibitors and analyzed their thermodynamic binding signatures. Our results provide a comprehensive and comparable data set of inhibitory action and mechanisms of JAK2 inhibitors and offer novel insights into the development of new drugs targeting JAK2 with improved potency and selectivity.

## Results

### Selectivity Analysis of JAK2 Inhibitors

The development and design of next-generation JAKinibs require detailed information on the JAK selectivity and binding modes of current clinical-stage compounds. Although individual JAK inhibitors are well characterized, compound potency and selectivity data are not directly comparable due to large interstudy variance in separate reports. In addition, atomic resolution structural data on clinical-stage drugs, which could guide future inhibitor development efforts, is lacking. Here, we performed a parallel, in vitro profiling of 10 JAK-inhibitors that have been designed for applications in MPN or other hematologic malignancies for binding to their kinase and pseudokinase domains and for inhibition of catalytic activity of JAK family members. We also determined the crystal structures of JAK2 with seven structurally diverse, clinical-stage JAK2 inhibitors, including recently approved pacritinib (Vonjo) and momelotinib (Ojjaara).

It was of interest to include the pseudokinase domain in the analysis since the MPN mutations, including V617F, concentrate in this region. Furthermore, the first drug targeting the pseudokinase ATP pocket, TYK2 inhibitor deucravacitinib, has been approved for the treatment of psoriasis. Most inhibitors did not show significant binding to pseudokinase domains, except for cerdulatinib, AT-9283 and pacritinib, which demonstrated one- or two-digit nanomolar affinities for the pseudokinase domains of JAK1 (cerdulatinib, AT-9283) and/or TYK2 (cerdulatinib, AT-9283 and pacritinib) (Table S1). Selectivity of the JAKinibs was profiled with binding and kinase activity assays. Based on the in vitro binding analysis, all compounds bind to JAK2 kinase domain at nanomolar affinity (≤30 nM) but also target other JAK family members at submicromolar affinity (Table 1). Pacritinib (≥6-fold selectivity for JAK2 over other JAKs) and fedratinib (≥20-fold selectivity) possessed the highest JAK2 selectivities in the binding assay, whereas other compounds demonstrated equal binding to two or more JAK family members. The compounds also inhibited the catalytic activity of JAK2 in vitro with low-nanomolar IC_50_ values, and the most potent inhibition was observed by ilginatinib, lestaurtinib, and ruxolitinib. The inhibition of other JAK family members varied between the compounds, and most JAKinibs were potent JAK1 inhibitors. Particularly, itacitinib demonstrated a more potent inhibition of JAK1 over JAK2 (Table 1). The highest JAK2 selectivity over JAK1 was demonstrated for pacritinib (26-fold selectivity over JAK1), momelotinib (13-fold), AT9283 (9-fold), ilginatinib (9-fold), and fedratinib (6-fold) (Figure S1).

**Table 1:** Binding and Activity Inhibition of JAK Inhibitors on JAK Family Members^a^

	binding to kinase domain *k*_d_ [nM]				activity inhibition IC_50_ [nM]			
	JAK1	JAK2	JAK3	TYK2	JAK1	JAK2	JAK3	TYK2
ruxolitinib	19**	0.8	27**	1.1	5.8	3.2	278****	60***
momelotinib	72****	6.7	19***	9.2	143****	11	325****	252****
ilginatinib	16	4.5	19	21	7.1****	0.8	182****	216****
itacitinib	51**	26	324**	385**	3.4****	54	10 799****	4095****
cerdulatinib	23*	4.8	3.3	6.9	22	11	25	26*
pacritinib	318****	6.6	41***	125****	371****	14	311****	536****
lestaurtinib	2.8	2.9	1.1*	7.3	3.5	3.0	2.9	15****
gandotinib	8.2	11	13	16	7.3	6.2	199****	92****
fedratinib	258****	4.3	85****	217****	56***	10	1812****	3714****
AT9283	37***	3.3	6.6	12*	65****	7.8	17	36***

a Data is derived from FP binding or Lance Ultra kinase assays and presented as average of triplicate samples from three individual experiments. Numbers are the nanomolar binding affinity (*k*_d_) or half-maximal inhibitory concentrations (IC_50_) of a JAKinib for JAK family members. One-way analysis of variance with Dunnett’s multiple comparison post hoc test was used for assessment of statistically significant differences in *k*_d_ or IC_50_ of JAK-family members in comparison to JAK2. Statistical significance is indicated by stars: *p < 0.0332, ** <0.0021, *** <0.0002, and **** <0.0001.

### Structural Analysis of JAK2 Inhibitor Binding

We determined the crystal structures of seven JAK2-inhibitor complexes to understand what drives the potency and selectivity of these compounds. No structural information on the compounds in complex with kinases is available, except for momelotinib. Two momelotinib structures can be found in PDB database (https://www.rcsb.org/), one in complex with Unc-51-like kinase 3 (ULK3) (PDB ID 6FDZ) and the other with activin receptor-like kinase-2 (ALK2) (PDB ID 7NNS). Although the conformation of momelotinib is similar in JAK2 and ULK3 structures, the compound in ULK3 is shifted significantly toward the G-loop resulting in >3 Å difference in the position of the, respective, nitrile groups of momelotinib. This is likely caused by the difference in the conformation of the DFG-motif phenylalanine in the structures. In ALK2 and JAK2, momelotinib superposes well at the hinge region but changes conformation deeper in the pocket leading to >5 Å difference for the nitrile groups of the compound.

Structural analysis showed that all compounds anchor to the ATP-pocket of the kinase domain of JAK2 by making two or three hydrogen bonds with the hinge region. However, the compounds showed distinct additional hydrogen bonding interactions with several critical residues in the G-loop, near the catalytic loop, and in the β1-strand. The ATP pocket itself displayed no major conformational changes to accommodate the inhibitors, except for the G-loop, which is highly mobile in protein kinases and controls both ATP binding and phosphoryl transfer to substrate proteins.^21^

**Figure 2:**
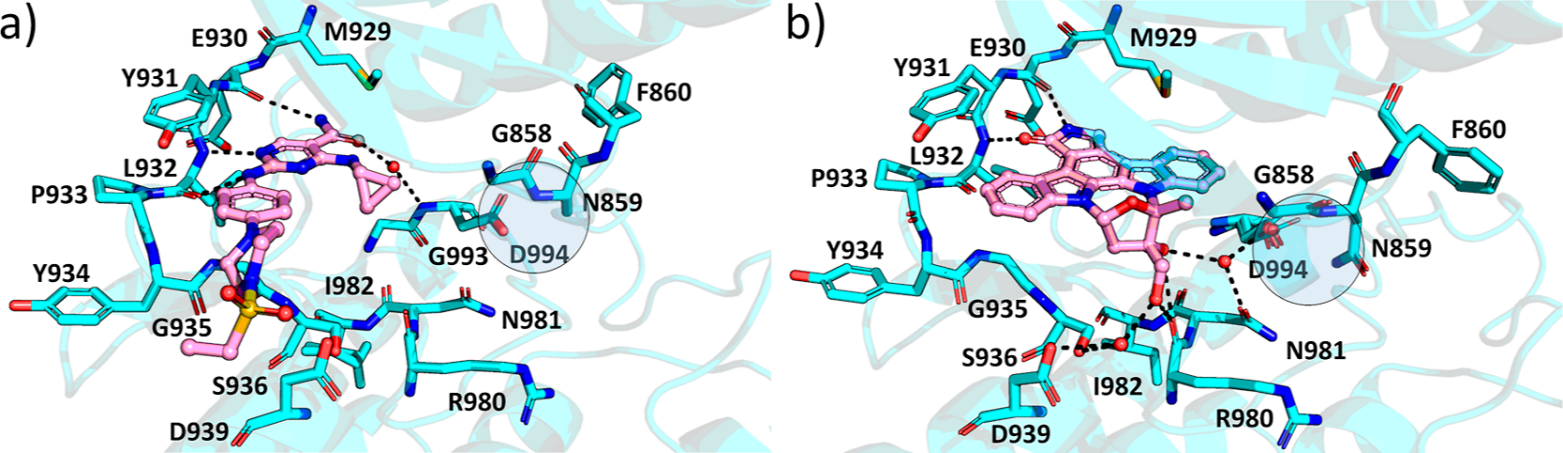
Binding modes of cerdulatinib and lestaurtinib to JAK2. (a) JAK2–cerdulatinib complex and (b) JAK2–lestaurtinib complex. JAK2 is shown in cyan, and the inhibitor is shown in pink. Water molecules directly binding to the inhibitor are shown as red spheres. Hydrogen bonds are depicted as black dotted lines. The regions with Asn859 and Asp994 are highlighted.

Cerdulatinib and lestaurtinib displayed potent inhibition of all JAK family members (Table 1). Binding of these pan-JAK inhibitors stabilizes a closed G-loop structure, where Asn859 from the G-loop and Asp994 from the DFG motif are within hydrogen-bonding distance (Figures 2 and S2). Cerdulatinib makes three hydrogen bonds to the hinge region: two to main chain carbonyl oxygens of Glu930 and Leu932 and one to main chain amide of Leu932. Cerdulatinib also engages in a water-mediated hydrogen bond with Asp994. Lestaurtinib forms hydrogen bonds with the carbonyl oxygen of Glu930 and the backbone amide of Leu932 at the hinge and also makes a direct hydrogen bond with Arg980 next to the catalytic loop and water-mediated hydrogen bonds to Asp994, Asn981, Ser936, and Asp939.

**Figure 3:**
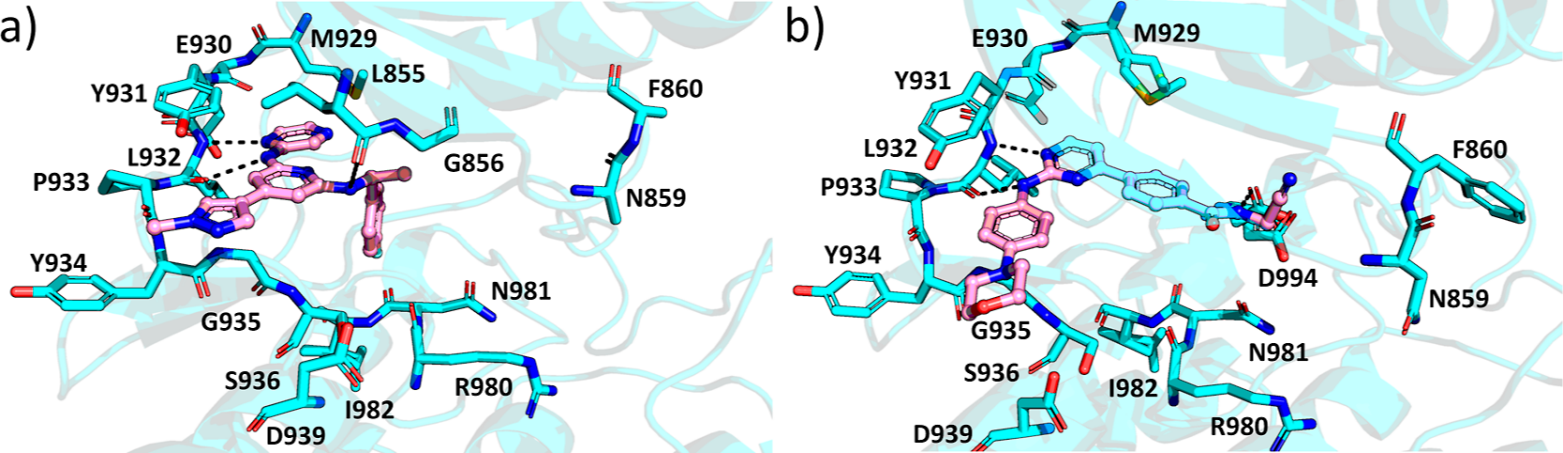
Binding modes of ilginatinib and momelotinib compared to JAK2. (a) JAK2–ilginatinib complex and (b) JAK2–momelotinib complex. JAK2 is shown in cyan and the inhibitor in pink. Water molecules directly binding to the inhibitor are shown as red spheres. Hydrogen bonds are depicted as black dotted lines.

In a more open G-loop conformation induced by JAK2-selective ilginatinib and momelotinib, the Asn859-Asp994 interaction is broken (Figure 3 and Figure S3). Both compounds form hydrogen bonds to backbone carbonyl and amide of Leu932 at the hinge region and make one additional hydrogen bond outside the hinge, ilginatinib with Leu855 from the β1-strand and momelotinib with Asp994 from the DFG motif.

**Figure 4:**
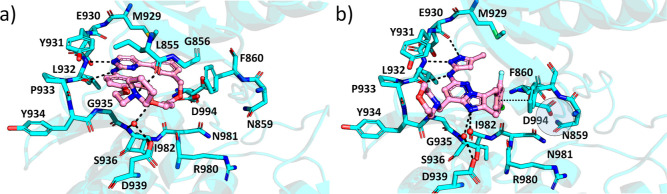
Binding modes of pacritinib and gandotinib to JAK2. (a) JAK2–pacritinib complex and (b) JAK2–gandotinib complex. JAK2 is shown in cyan and the inhibitor in pink. Water molecules directly binding to the inhibitor are shown as red spheres. Hydrogen bonds and π-stacking interactions are depicted as black dotted lines. The regions with Asn859 and Asp994 are highlighted.

Pacritinib and fedratinib possessed the highest JAK2 selectivity in the binding assay. Structure of the JAK2-fedratinib complex has been previously determined.^22^ Fedratinib binds to the ATP pocket with the G-loop in an open conformation akin to that of JAK2-selective momelotinib and does not engage in additional hydrogen bonding with the binding site apart from the hinge region. Pacritinib makes two hydrogen bonds to the hinge, to the main chain carbonyl and amide of Leu932, and a hydrogen bond with Leu855 from the β1-strand. The binding of pacritinib is also stabilized by water-mediated hydrogen bonds to Ser936 and Asp939. Pacritinib stabilizes a collapsed G-loop structure in JAK2, where Phe860 is buried within the binding site (Figures 4a and S4). Interestingly, this G-loop conformation can also be found in the gandotinib structure, where it is further stabilized by a π-stacking interaction with the compound via Phe860 (Figures 4b and S4). Gandotinib binds to the hinge with three hydrogen bonds, one to main chain carbonyl of Glu930 and two to main chain carbonyl and amide of Leu932. In addition, gandotinib forms water-mediated hydrogen bonds to Ser936 and Asp939. Despite the collapsed G-loop, gandotinib does not display a markedly high JAK2-selectivity. Pacritinib and gandotinib represent the first JAK2 structures with a collapsed G-loop conformation.

**Figure 5:**
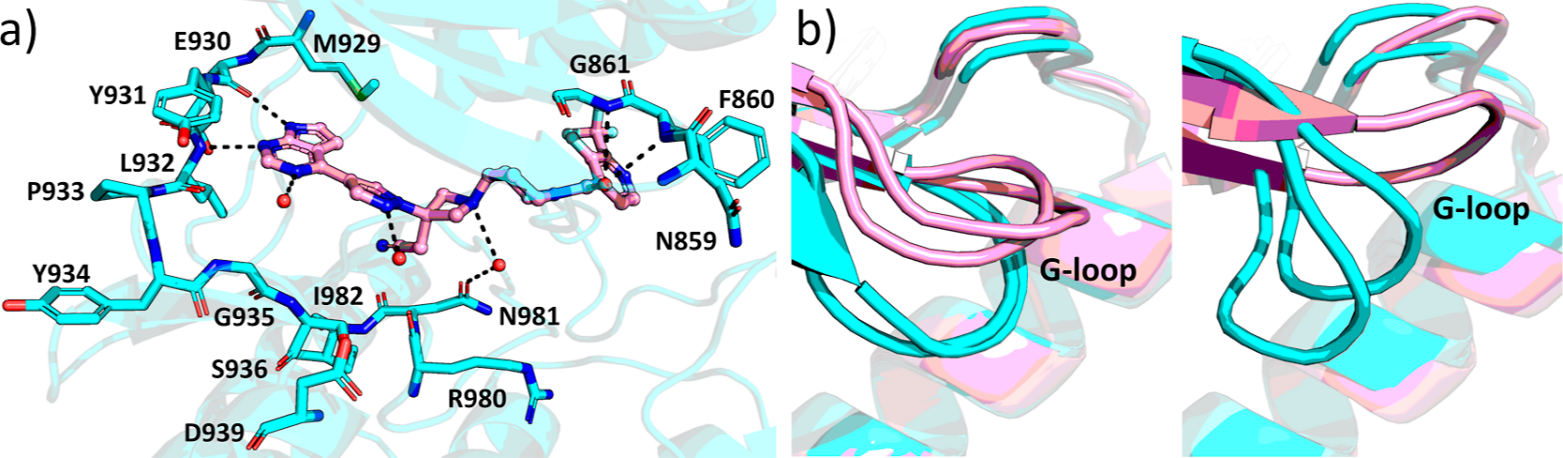
Binding mode of itacitinib and G-loop conformations of the JAK2–inhibitor complexes. (a) JAK2–itacitinib complex. JAK2 is shown in cyan and the inhibitor in pink. Water molecules directly binding to the inhibitor are shown as red spheres. Hydrogen bonds are depicted as black dotted lines. (b) G-loop conformations in the crystal structures. On the left: superposition of lestaurtinib and cerdulatinib (closed G-loop structure, cyan) to ilginatinib and momelotinib (open G-loop structure, pink). On the right: superposition of pacritinib and gandotinib (collapsed G-loop structure, cyan) to itacitinib (extended G-loop structure, pink).

Structural analysis demonstrated that the binding mode of JAK1-selective itacitinib differs from the other compounds: it binds to the pocket with the G-loop in an extended conformation reaching the tip of the G-loop, where it forms hydrogen bonds to backbone amides of Phe860 and Gly861 (Figure 5a and Figure S5). Other inhibitors do not extend to the tip of the G-loop. In addition, itacitinib makes a water-mediated hydrogen bond to Asn981. At the hinge, itacitinib forms hydrogen bonds to backbone carbonyl of Glu930 and backbone amide of Leu932.

**Figure 6:**
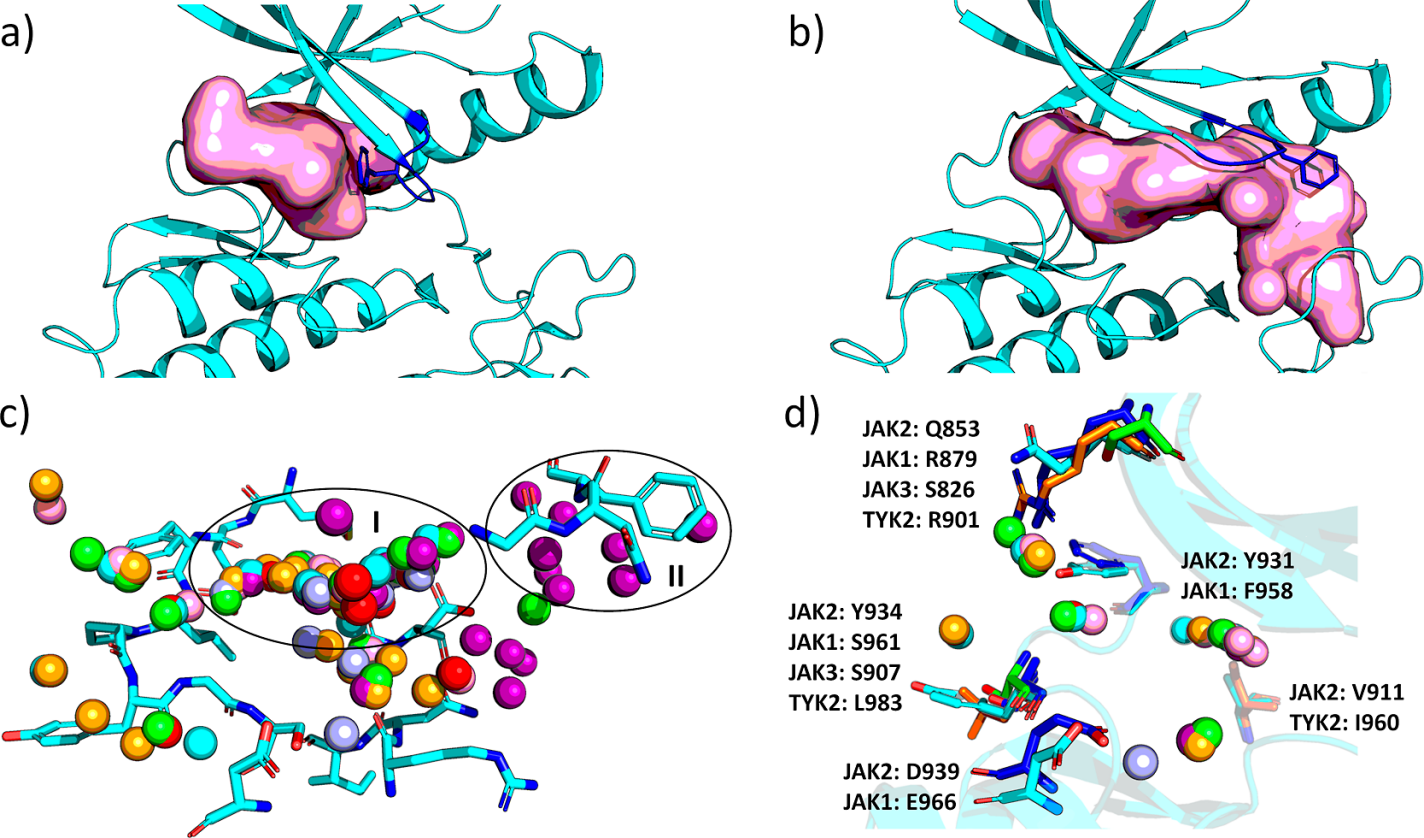
Binding pocket analysis. Binding pocket volumes of the JAK2–gandotinib (a) and JAK2–itacitinib (b) complexes. G-loop movement results in large changes in binding pockets. JAK2 is shown in cyan, the pocket in pink, and the G-loop in blue. Binding pocket analysis was done with SiteMap.^23^ (c) All waters from the seven complexes based on WATsite analysis with Δ*G* ≥ 2 kcal/mol were superposed on itacitinib structure. Two high-energy water clusters were identified: (I) main high-energy water cluster for all complexes and (II) itacitinib-specific high-energy water cluster at the tip of the G-loop. Water molecules are colored red (pacritinib), green (momelotinib), light blue (lestaurtinib), purple (itacitinib), cyan (ilginatinib), orange (gandotinib), and pink (cerdulatinib). d) Binding site differences influencing ATP-pocket solvation and thermodynamics. Only simulated waters close to nonconserved residues are shown. JAK2 is colored cyan, JAK1 blue, JAK3 green, and TYK2 in orange.

Taken together, most JAK inhibitors primarily designed for targeting MPN potently inhibited the catalytic activity of JAK1, whereas inhibition of JAK3 and TYK2 was generally less effective. JAK2 selectivity was shown by pacritinib, momelotinib, AT9283, ilginatinib, and fedratinib. The structural determinant, which aligns with JAK2 selectivity, is a G-loop conformation where Asn859 from the G-loop and Asp994 from the DFG motif are not in the hydrogen-bonding distance. This occurs in JAK2-selective ilginatinib, momelotinib (Figures 3 and 5b), and fedratinib, which bind the G-loop in an open conformation and also in pacritinib (Figures 4a and 5b), where the G-loop is collapsed. Despite the collapsed G-loop in the gandotinib structure, the Asn859-Asp994 hydrogen bond remains intact, and gandotinib displays low JAK2-selectivity. Asn859 is not conserved in JAK1 and TYK2, which have histidine at this position that might affect the plasticity of the G-loop and compound potency toward these family members. Interestingly, the collapsed G-loop conformation in pacritinib and gandotinib leads to ATP-pocket, which is fully closed in the direction of the G-loop (Figure 6a,b).

### Thermodynamics of Inhibitor Binding

To gain further insights into the binding mechanism of the inhibitors to JAK2, we analyzed the binding thermodynamics with isothermal titration calorimetry (ITC) (Table 2 and Figures S6 and S7). The inhibitors displayed comparable binding affinities, aligning with the results from binding and activity assays but distinct ligand binding mechanisms. The binding of momelotinib, ilginatinib, cerdulatinib, and lestaurtinib is mainly driven by enthalpy. A notable opposing entropy component with momelotinib is likely due to rigidification of the compound upon binding to the ATP pocket. Interestingly, the binding of pacritinib and gandotinib, which both form a collapsed G-loop, is driven both by enthalpy and entropy. The favorable entropy is partly due to the desolvation of Phe860 at the tip of the G-loop, which buries itself to the ATP-pocket upon inhibitor binding. The highly favorable binding entropy of itacitinib is likely linked to the displacement of three ordered water molecules (visible in the momelotinib crystal structure, Figure S8) from the hydrated cavity near the tip of the G-loop. To analyze the effect of pocket desolvation by the inhibitor binding more thoroughly, we performed molecular dynamics-based analysis of the binding pocket water structure and thermodynamics for all JAK2–inhibitor complexes (Table 2 and Figures 6c and S9). We observed two main clusters of high-energy water molecules in the simulations. The first water cluster superposed in the middle of the binding pocket and was shared by all inhibitors. A more sparsely populated second water cluster was located at the tip of the G-loop and is exclusive to itacitinib. Accordingly, the highest desolvation free-energy gain, arising from the displacement of waters upon inhibitor binding, was observed with compounds occupying the front pocket and not extending toward the G-loop or toward the solvent (lestaurtinib and gandotinib). Figure 6d shows the nonconserved residues around the ATP pocket in the JAK family, which might influence the water structure in different JAKs and impact compound selectivity.

**Table 2:** Inhibitor Binding and Protein Desolvation Thermodynamics^a^

	momelotinib	ilginatinib	itacitinib	cerdulatinib	pacritinib	lestaurtinib	gandotinib^b^
Experimental thermodynamical parameters from ITC							
Δ*G* (kcal/mol)	–11.3	–11.3	–10.7	–10.8	–10.9	–11.2	–10.7
Δ*H* (kcal/mol)	–14.4 ± 0.2	–12.5 ± 0.2	–5.4 ± 0.1	–10.4 ± 0.1	–7.6 ± 0.1	–12.1 ± 0.2	–4.1 ± 0.1
–Δ*T*S (kcal/mol)	3.2	1.2	–5.4	–0.4	–3.3	1.0	–6.6
*n*	0.54	0.84	1.25	0.51	0.66	0.65	
*K*_D_ (nM)	4.5 ± 18.6	4.0 ± 15.9	10.7 ± 62.9	8.9 ± 45.9	8.9 ± 40	4.9 ± 16.8	11
Computational solvation thermodynamics from water mapping							
Δ*G*_desolv_ (kcal/mol)	–21.4	–32.1	–33.0	–29.8	–26.2	–43.8	–49.0
Δ*H*_desolv_ (kcal/mol)	0.7	–2.3	2.4	–10.5	–3.2	–4.7	–15.2
–Δ*T*S_desolv_ (kcal/mol)	–22.2	–29.8	–35.4	–19.3	–23.0	–39.1	–33.8

a ITC measurements were performed at 21 °C with protein concentrations between 3 and 20 μM and inhibitor concentrations between 30 and 200 μM.

b *In vitro* thermodynamics analysis for gandotinib was done with FP measurements combined with ITC (see the Supporting Information).

### Cell-Based Potency of the Inhibitors

**Figure 7:**
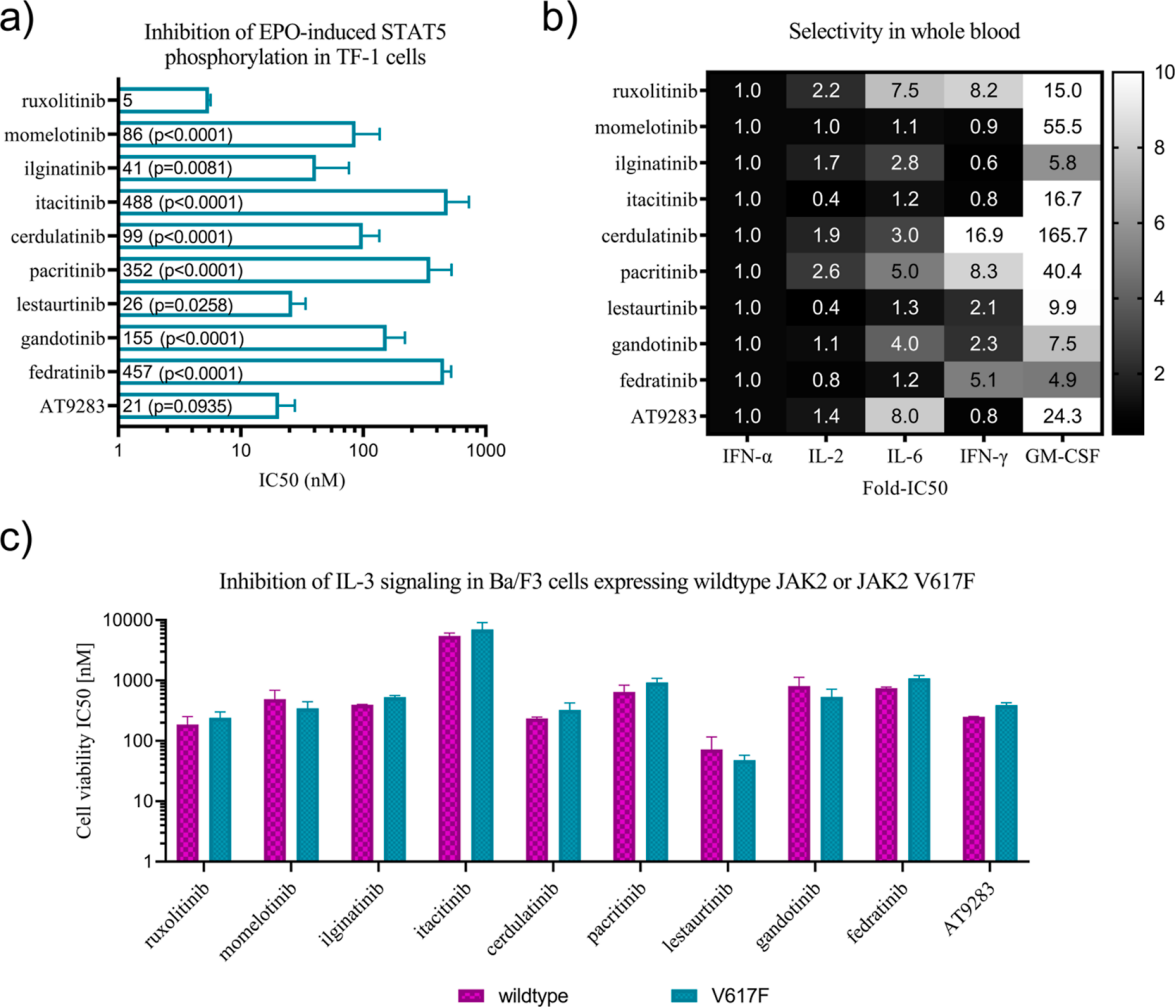
Cellular effects of the JAK inhibitors. (a) Inhibition of JAK2-mediated EPO-pSTAT5 signaling in TF-1 cells. The data are presented as mean nanomolar IC_50_ values. Error bars indicate standard deviation. Statistical significance was assessed by one-way analysis of variance with Tukey multiple comparison analysis, and p-values are shown relative to ruxolitinib. (b) Selectivity of JAK inhibitors for JAK-mediated cytokine signaling in whole blood. The data is presented as mean fold selectivity for each cytokine normalized to inhibition of IFNα, which was potently inhibited by all the inhibitors. IFN-α-pSTAT1 (JAK1/TYK2), IL-6-pSTAT3 (JAK1), IFN-γ-pSTAT1 (JAK1/JAK2), and GM-CSF-pSTAT5 (JAK2) inhibition was measured in CD33+ monocytes and IL-2-pSTAT5 inhibition was measured in CD4+ T cells. (c) Half-maximal inhibitory concentrations of JAKinibs for viability of MPN model cell lines Ba/F3-expressing human JAK2 wildtype or V617F. The data is an average of 4–6 measurements, and error bars indicate standard deviation.

To understand how the JAK selectivity of JAKinibs is translated into inhibition of JAK-mediated cytokine signaling in cells, we profiled the inhibitors in the erythroblast cell line TF-1 or in whole blood upon stimulation with EPO (JAK2), GM-CSF (JAK2), IFN-α (JAK1/TYK2), IFN-γ (JAK1/JAK2), IL-2 (JAK1/JAK3), and IL-6 (JAK1). All the tested JAKinibs inhibited JAK2-mediated EPO-pSTAT5 signaling in TF-1 cells with variable submicromolar IC_50_ values, and the most potent inhibition was exhibited by ruxolitinib, AT9283, lestaurtinib, and ilginatinib (Figure 7a). The inhibition of EPO-induced STAT5 activation is not completely in line with the effects of inhibitors on in vitro activity, e.g., ilginatinib harbors the lowest IC_50_ for JAK2 activity inhibition while being less effective in EPO inhibition than ruxolitinib. Also, fedratinib seems to inhibit EPO signaling poorly compared to ruxolitinib. Multiple factors might contribute to this, e.g., cell permeability or drug elimination processes might vary depending on the inhibitor being considered.

GM-CSF and IFN-γ are canonical JAK2 or JAK1-/JAK2-mediated cytokine pathways. JAK1-/JAK2-mediated IFN-γ was the primary inhibited cytokine among the cytokines tested with momelotinib, ilginatinib, itacitinib, and AT-9283, whereas inhibition of GM-CSF signaling was weak (Figure 7b). Cytokine selectivity studies in whole blood indicated that despite strong inhibition of JAK2 catalytic activity in vitro, the MPN-approved JAKinibs inhibited primarily the JAK1-mediated cytokine signaling routes IL-2 (JAK1/JAK3), IL-6 (JAK1), IFN-α (JAK1/TYK2), and IFN-γ (JAK1/JAK2) (Figure 7b).

To compare cytokine inhibition in blood with the inhibition of EPO-induced STAT5 signaling in TF-1 cells, the effect of inhibitor binding to plasma proteins was excluded by calculation of unbound IC_50_ values for the whole blood results (unbound fraction data was not available for ilginatinib, gandotinib, and AT9283). The IC_50_ values of EPO-induced STAT5 signaling were clearly lower than the unbound IC_50_ values for GM-CSF but generally at similar range with the values for IFN-γ (Table S2). EPO is one of the primary inhibited cytokine pathways for ruxolitinib, momelotinib, and lestaurtinib (Table S2). JAK1-selective itacitinib demonstrated a two-digit nanomolar unbound IC_50_ for IFN-γ (JAK1/JAK2), whereas inhibition of EPO (JAK2) signaling was lower with a three-digit nanomolar IC_50_.

To further analyze the connection between in vitro JAK2 binding or activity inhibition with inhibition of cytokine signaling, we performed correlation analyses between the cellular cytokine signaling IC_50_ values and in vitro JAK2 kinase domain binding or JAK2 activity IC_50_ values for the hematological JAKinibs. We also included 10 rheumatic disease-evaluated JAKinibs,^24^ which showed pan-JAK-, JAK1-, JAK3-, or TYK2-selective inhibition in the analysis (cytokine IC_50_ data in Table S3). Binding affinity to the JAK2 kinase domain did not correlate with the inhibition of JAK2-mediated cytokine signaling (data not shown), whereas in vitro inhibition of JAK2 activity correlated well with EPO and IFN-γ inhibition but not with GM-CSF (Table S4). Therefore, in vitro activity inhibition describes the cellular effects better than the kinase domain-binding affinity. Furthermore, the inhibition of GM-CSF, although canonically mediated by JAK2, might not be a direct measure for cellular JAK2 inhibition.

The selectivity of the MPN-evaluated JAKinibs toward JAK2 V617F, the most frequent gain-of-function mutation in MPN patients, was studied in the IL-3-dependent cell line Ba/F3, a well-established model system for cytokine receptor studies. Ba/F3 cells were engineered to express either wild-type human JAK2 or human JAK2-V617F and the endogenous mouse Jak2 genes were inactivated by CRISPR/Cas9. Experiments were performed in the presence of IL-3, which is required for the growth of cells expressing wild-type JAK2. The inhibitory effect of JAKinibs on the viability of Ba/F3-hJAK2 cells was submicromolar, except for the JAK1-selective itacitinib that resulted in micromolar IC_50_ values. Most potent inhibition of the humanized Ba/F3 cells was demonstrated by lestaurtinib and ruxolitinib. The current MPN-indicated JAKinibs did not show selectivity for JAK2-V617F over wild-type JAK2 (Figure 7c).

## Discussion and Conclusions

JAK family members have distinct roles in cellular functions.^25^ JAK1 is vital for the development and function of the immune system, and JAK2 plays a critical role in the formation of myeloid and erythroid blood cells but also mediates immune functions together with other JAKs. JAK3 plays an important role in regulating the function of the immune system, and TYK2 mediates immune functions via the regulation of T- and natural killer cells. Hyperactive JAK2 signaling is driving MPN pathogenesis,^26^ and the development of new JAK inhibitors aims at increasing JAK2 selectivity. Recently Kong et al.^27^ reported a comprehensive profiling of ruxolitinib, pacritinib, fedratinib, and momelotinib that revealed important insights into their differential cellular, RNA, and signaling effects including modulation of iron levels. Here, we performed a head-to-head profiling for MPN-targeted JAKinibs, which demonstrated variance in the selectivity profiles of the JAKinibs from moderate JAK2-selectivity (pacritinib, momelotinib, ilginatinib, AT-9283, and fedratinib) to JAK1 selectivity (itacitinib) or JAK1–JAK2 targeting (ruxolitinib and gandotinib). Lestaurtinib and cerdulatinib were potent inhibitors of all JAK family members, whereas the inhibitory potential of other JAKinibs for JAK3 and TYK2 was low.

Dimerization of JAKs in five known combinations (JAK1/JAK2, JAK1/JAK3, JAK1/TYK2, JAK2/TYK2, and JAK2/JAK2) is required in JAK-mediated cytokine signaling. However, depending on the cytokine pathway being considered, the activity of both JAK family members may not be equally required for signal transduction. Therefore, the prediction of cytokine signaling effects of a JAKinib based on in vitro JAK selectivity profile is complex and in vitro inhibition of JAK activity does not necessarily correlate with cytokine inhibition. The cytokine receptors selected for this study employ different JAK combinations except for the GM-CSF and EPO, which canonically signal through JAK2. We observed inhibition of EPO and JAK1/JAK2-mediated IFN-γ but not GM-CSF to correlate with in vitro inhibition of JAK2 activity. The reason for this is not fully understood, but EPO and GM-CSF receptors vary in their structure, with the GM-CSF receptor demonstrating a dodecameric complex present in granulocytes and monocytes, whereas the EPO receptor is a dimer present in myeloid lineage cells. JAK1 might affect GM-CSF signaling as genetic models have revealed JAK1 dependency for IL-3 signaling that uses the same bc-receptor chain as GM-CSF.^28^

Hyperactivating JAK2 V617F mutation located in the pseudokinase domain of JAK2 is predominant in MPNs and is found in 95% of patients with PV and 50% of patients with ET and PMF.^29^ None of the inhibitors demonstrated V617F-specific inhibition in Ba/F3 cell assays (Figure 7c), which supports previous conclusions that the current JAKinibs do not show selectivity for the JAK2 V617F mutation.^30,31^ Gandotinib has been reported to be selective for JAK2 V617F over the wild-type JAK2 (24-fold) in Ba/F3 cell proliferation assays.^32^ Our data shows that IC_50_ for hJAK2-V617F cells is numerically lower than that for wild-type cells (1.5-fold selectivity). The selectivity difference may result from the different cell models, Ba/F3 cells engineered to carry human JAK2 (our study) vs murine homologue, or from other experimental conditions (e.g., starvation or stimulation conditions). As the V617F mutation is in the regulatory pseudokinase domain, mutant selectivity might not be possible to achieve with type-I inhibitors, which target the active conformation of the ATP pocket of the kinase domain. Interestingly, Incyte recently presented preliminary preclinical results as an American Society of Hematology (ASH) conference abstract of INCB160058, which is a pseudokinase domain, ATP-pocket targeting JAK2 inhibitor that demonstrated V617F selectivity in cells.^33^ Mechanistically, this inhibitor functions differently than kinase domain-targeted compounds as it appears to inhibit receptor dimerization. Still, type-I compounds are well-validated in the clinic, and improving JAK2 selectivity is an important goal for their future development.

**Figure 8:**
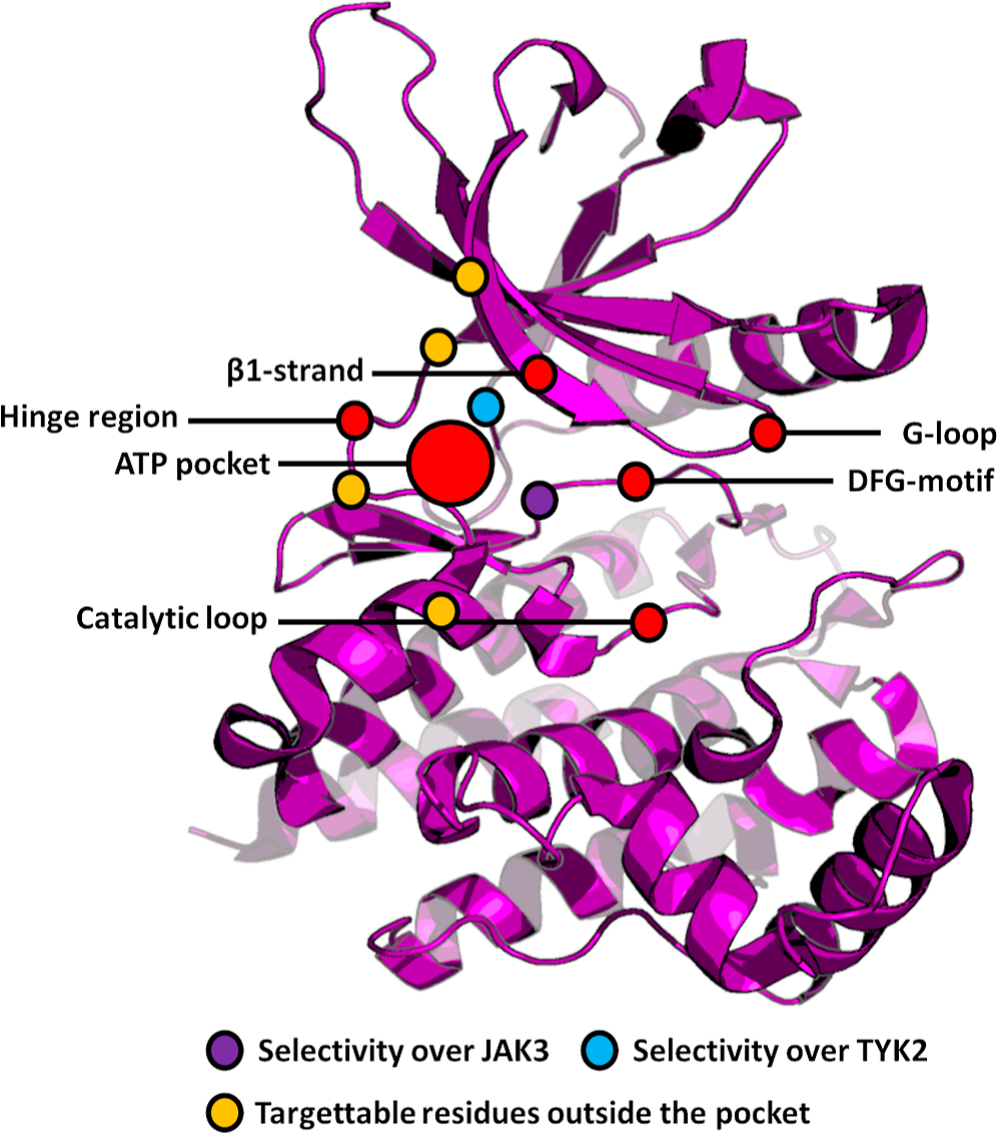
Key targets in the JAK2 kinase domain for JAK2-selective inhibitor design.

A prominent feature of many JAK inhibitors, such as ruxolitinib, tofacitinib, baricitinib, and upadacitinib, is their extension toward the G-loop. The differences in the G-loop region have been used as a basis for improving selectivity during inhibitor design.^34^ Our data indicate that high JAK2 selectivity can be achieved by intentionally not extending the inhibitors along the G-loop (Figure 8). For high potency, binding solely to the front pocket is sufficient. Additional interactions can be gained from the β1-strand (pacritinib) or the DFG motif (momelotinib). Extending compounds outside the pocket toward Asp939 could have negative effects on the selectivity over JAK1^35^ where a larger glutamate is found at this position. Gandotinib, pacritinib, and lestaurtinib, which make a water-mediated hydrogen bond to Asp939, could directly interact with the glutamate in this position in JAK1. In line with this concept, gandotinib and lestaurtinib showed no selectivity over JAK1.

High selectivity over JAK3 and TYK2 is achieved by most of the inhibitors tested. Selectivity over JAK3 can be gained by extending the compound toward Gly993 (alanine in JAK3). Gandotinib, itacitinib, and ilginatinib take advantage of this as the compounds clash with the alanine in JAK3. A region in the back pocket below the methionine gatekeeper harbors Val911 in JAK2 but isoleucine (Ile960) in TYK2. This slightly larger residue clashes with hinge-binding ring moieties in most structures. The exceptions to this are cerdulatinib, which has an amide in this position, allowing more rotational freedom, and gandotinib, which has a methyl group at the same position. JAK selectivity can also be influenced by ATP-pocket solvation, which is influenced by subtle differences in the binding site. While differences in the active site residues explaining inhibitor selectivity are scarce, residues in the second or third coordination shell show less conservation and can affect the water structure and desolvation by inhibitor binding. For example, Gln853 in JAK2 is replaced by arginine in JAK1 and TYK2 and by serine in JAK3, disrupting the nearby high-energy water cluster (Figure 6d). At the hinge region, the major nonconserved residue in JAK2 is Tyr934, which is replaced by serine in JAK1 and JAK3 and leucine in TYK2. Although the side chains of the residues point outward from the ATP pocket, they can affect the network of waters displaced by inhibitor binding. In addition to this, Tyr931 is replaced by phenylalanine in JAK1 similarly affecting the pocket solvation. At the back pocket, Val911 is replaced by isoleucine in TYK2 possibly affecting the nearby water cluster. These differences can explain variations in inhibitor potencies that occupy only the highly conserved front region of the ATP pocket.

In conclusion, current JAK2 inhibitors have limited selectivity and cannot distinguish between the wild-type and mutant forms of JAK2. Selective JAK2 inhibitor design should focus more on the differences in the second and third coordination shell and water-mediated effects as the high conservation of the ATP pocket does not provide many avenues for selective inhibitor development.

**Figure 9:**
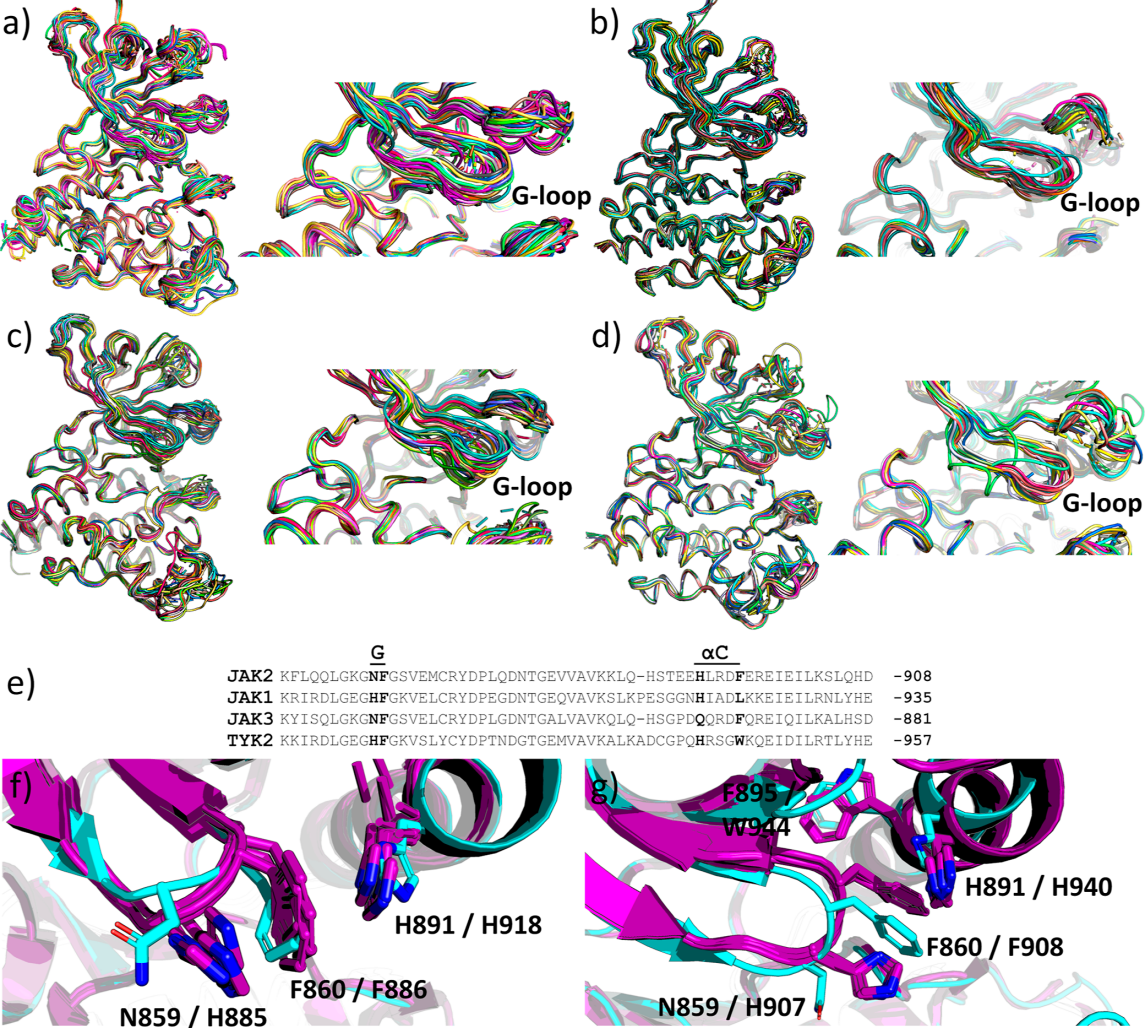
G-loop stability in JAKs. Superposition of all PDB coordinates (https://www.rcsb.org/, accessed 2/2023) of JAK2 (a), JAK1 (b), JAK3 (c), and TYK2 (d). A more stable G-loop can be seen in general in JAK1 and TYK2 than in JAK2 and JAK3. Close-up views of the G-loops are on the right. (e) Sequence alignment showing G-loop (G) and αC-helix (αC) regions in JAKs. Asn at the tip of the G-loop in JAK2 and JAK3 is replaced with His in JAK1 and TYK2. Histidine can stabilize the loop structure by forming stabilizing interactions with the αC-helix. (f) In JAK1, a sandwich His–Phe (G-loop)-His (αC-helix) stacking interaction can be seen, e.g., in pdb codes 6N7A, 6N7B, 6N7C, and 6N7D. In JAK2 structure with a similar G-loop conformation (JAK2–itacitinib complex), the G-loop is not stabilized with similar stacking interactions. (g) In TYK2, a T-shaped His–Phe (G-loop) stacking in the tip of the G-loop can be seen in certain structures, e.g., in pdb codes 4GJ2, 4GJ3, 4GII, and 4GIH. The stabilized phenylalanine is positioned against the αC-helix in a hydrophobic environment. Compared to a similar G-loop structure in the JAK2–cerdulatinib complex structure, no interaction with the tip of the G-loop and αC-helix is found. JAK1 and TYK2 are colored purple, and JAK2 in cyan.

There seems to be no clear advantage in extending the compounds toward and targeting the flexible G-loop for potency or JAK2 selectivity. Highly potent compounds can be developed that only target the front pocket, and the collapsed conformation of the G-loop is a good starting point for inhibitor design offering a stable, restricted binding cavity. This conformation is complemented with a favorable desolvation entropy of the G-loop and the water cluster coupled with a stabilizing π-stacking interaction potential with Phe860. Notably, the G-loop seems to be more stable in JAK1 and TYK2 due to stabilizing interactions with the αC-helix. Figure 9a-d shows the superposition of all of the, respective, JAK structures. The G-loop appears to be more stable in JAK1 and TYK2 compared to JAK2 and JAK3. Interestingly, the asparagine residue at the tip of the G-loop in JAK2 and JAK3 is replaced with histidine in JAK1 and TYK2 (Figure 9e). Histidine can stabilize the loop structure by forming stabilizing interactions with the αC-helix. In JAK1, a sandwich His885-Phe886 (G-loop)—His918 (αC-helix) stacking interaction can be seen in several crystal structures (Figure 9f). In the JAK2–itacitinib complex, which has a similar G-loop conformation, the G-loop is not stabilized with similar stacking interactions. In TYK2, a T-shaped His907-Phe908 (G-loop) stacking in the tip of the G-loop can be seen in certain structures (Figure 9g). The stabilized Phe908 is positioned against the αC helix in a hydrophobic environment. Compared to a similar G-loop structure in JAK2–cerdulatinib complex structure, no interaction with the tip of the G-loop and αC-helix is found. Taken together, the collapsed G-loop conformation could have a selectivity benefit toward JAK2 due to a more rigid G-loop structure in JAK1 and TYK2, as demonstrated by pacritinib, which displays the highest selectivity over JAK1 of the tested inhibitors.

## Experimental Section

### Inhibitors

Inhibitors were acquired from Cayman Chemical Co. [lestaurtinib (≥98%)], MedChemExpress [(itacitinib (99.97%) and ilginatinib (99.53%)], and Selleck Chemicals [(pacritinib (99.94%), gandotinib (99.95%), cerdulatinib (99.80%), momelotinib (99.75%), fedratinib (99.99%), AT9283 (100%), and ruxolitinib (99.98%)].

### Protein Expression and Purification

Expression and purification of recombinant human JAK1 JH1 D1003N (866–1154-His), JAK1 JH2 (553–856-His), JAK1 JH2-JH1 (553–1154-His), JAK2 JH1 (836–1132-His), JAK2 JH2 (503–827-His), JAK2 JH2-JH1 (513–1132-His), JAK3 JH1 (811–1124-His), JAK3 JH2 (511–790-His), JAK3 JH2-JH1 (511–1124-His), TYK2 JH1 (886–1187-His), TYK2 JH2 (564–876-His), and TYK2 JH2-JH1 (564–1187-His) were performed as described previously.^24^

Human JAK2 kinase domain (amino acids 840–1132) for structural studies and calorimetry was cloned in pFB-LIC-Bse vector (pFB-LIC-Bse was a gift from Opher Gileadi (Addgene plasmid #26108)^36^ with an N-terminal 6xHis-tag, and protein expression was done using baculovirus expression in High Five cells. Cells were suspended in lysis buffer [50 mM Tris (pH, 8.0), 500 mM NaCl, 10% glycerol, 0.5 mM TCEP, and 20 mM imidazole] and lysed by a freeze/thaw method. Protein was purified with IMAC using Protino Ni-NTA and eluted in a lysis buffer with 250 mM imidazole. The protein was further purified with HiLoad 16/600 Superdex 75 pre-equilibrated with 20 mM Tris pH 8.5, 300 mM NaCl, 10% glycerol, and 0.5 mM TCEP.

### Fluorescence Polarization Binding Assay

Binding of inhibitors to recombinant JAK JH1 (JAK1 JH1 D1003N and JAK2/3/TYK2 JH1) and JH2 (JAK1/2/3/TYK2 JH2) was assessed in the fluorescence polarization (FP) assay as described previously.^24^*K*_d_ values for JAK inhibitors were calculated as previously reported,^24^ and one-way analysis of variance (ANOVA) with Dunnett’s multiple comparisons post hoc test was used for assessment of statistically significant differences in *K*_d_ values of JAK family members in comparison to JAK2.

### Kinase Assay

Effect of inhibitors on kinase activity of recombinant JAK JH2-JH1 proteins was determined using the LANCE Ultra kinase assay (PerkinElmer) as described earlier.^24^ Phosphorylation of the tyrosine kinase substrate was monitored by measuring FRET (Ex. 320 nm, Em. 665 nm) in 5 min interval for 30 min using EnVision Multilabel Plate Reader (PerkinElmer), and IC_50_ values were obtained by fitting the slope of signal increase in function of time against inhibitor concentration using GraphPad Prism 9 with the “log(inhibitor) vs response (three parameters)” model. Fold-IC_50_ values were calculated inhibitor-wise by dividing IC_50_ for each JAK by IC_50_ for the JAK2. Data presented are averages of triplicate samples and from three individual experiments. ANOVA with Dunnett’s multiple comparison post hoc test was used for assessment of statistically significant differences in pIC_50_ of JAK family members in comparison to JAK2.

### EPO Signaling Assay

TF-1 cells^37^ were cultured in RPMI-1640 supplemented with 10% fetal bovine serum (FBS), 1% pen-strep, 1% glutamine, and 2 ng/mL human GM-CSF. Cells were starved overnight in RPMI-1640 supplemented with 0.6% FBS, 1% pen-strep, and 1% glutamine, after which cells were collected by centrifugation and resuspended in phosphate-buffered saline (PBS). TF-1 aliquots (20 μL; 90 000 live cells) were incubated with JAKinibs (concentration range of 0.1 nM–10 μM) or dimethyl sulfoxide (vehicle control) in 96-deep-well plates at 37 °C for 60 min, after which the samples were incubated with 100 ng/mL erythropoietin (Peprotech) or PBS (unstimulated control) at 37 °C for 15 min. Samples were fixed by the addition of paraformaldehyde to a final concentration of 1.6% and incubation at RT for 10 min. Samples were washed twice with PBS, permeabilized with methanol, and stored −80 °C for up to 1 week. Samples were fluorescent barcoded in sets of 15 samples using Pacific Orange and Pacific Blue NHS esters at different combinations of concentrations of 0/0.15/1.35/7.5 μg/mL, after which a set of 15 samples were combined for staining with the PE-conjugated pSTAT5 antibody (BD) for 30 min at room temperature protected from light. After two washes with PBS supplemented with 0.1% bovine serum albumin and 0.01% NaN3, the samples were analyzed by using a CytoFLEX S flow cytometer (Beckmann Coulter). Data analysis was performed using FlowJo software (v10.7.1). Live cells were gated, the 15 individual samples were separated in a sample set based on intensities of barcoding dyes, PE fluorescence histograms were created, and the median fluorescence intensity (MFI, arithmetic median) was noted. IC_50_ values were obtained by fitting median fluorescence intensity against inhibitor concentration in Graphpad Prism. ANOVA with Tukey multiple comparison post hoc test was applied for assessment of statistically significant differences in pIC_50_ between JAK inhibitors.

### Whole-Blood Cytokine Signaling Assay

To study the effects of JAKinibs on cytokine signaling in whole blood, human peripheral blood was collected from healthy voluntary donors (*N* = 3) who gave informed consent for the study. All research with human subjects was carried out in compliance with the Helsinki Declaration and according to protocols approved by the Tampere University Hospital Ethics Committee. Reagents, assay parameters, and data analysis were performed as described earlier,^24^ unless otherwise stated. Briefly, blood was incubated (1 h, 37 °C) with inhibitors (concentration range of 0.1 nM–10 μM) followed by incubation (15 min, 37 °C) either with PBS, IFN-α, IFN-γ, IL-2, IL-6, or GM-CSF. After subsequent fixation and lysis of red blood cells, cells were permeabilized, fluorescent barcoded, stained for surface markers and pSTATs, and analyzed by FACSAria fusion flow cytometer (BD). Samples were identified from a sample set based on intensities of barcoding dyes, CD4+ T cells and CD33+ monocytes were gated, and MFIs at PE- (pSTAT3), AF488- (pSTAT1), and AF647-channels (pSTAT5) were calculated for each cell population. If cytokine stimulation induced pSTAT-signal increase ≥50%, inhibition parameters were calculated. IC_50_ values were obtained by fitting pSTAT MFI against inhibitor concentration using “log(inhibitor) vs response (three parameters)” model in GraphPad Prism 9. Fold-IC_50_ values were calculated compound-wise for canonical cytokine-pSTAT-pairs (IL-2, pSTAT5; IL-6, pSTAT3; IFN-a pSTAT1; IFN-g, pSTAT1; and GM-CSF, pSTAT5) by dividing IC_50_ values with IC_50_ of IFN-α.

Plasma protein binding of the inhibitors was considered by calculating unbound IC_50_ values (IC50_u_), if unbound fraction (*f*_u_) data was available
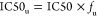


Unbound fraction values were as follows: ruxolitinib 0.03,^38^ itacitinib 0.26,^39^ fedratinib 0.09,^40^ pacritinib 0.01,^41^ momelotinib 0.19,^42^ cerdulatinib 0.22,^43^ and lestaurtinib 0.01.^44,45^ All data presented are averages of three individual experiments.

Pearson correlation analysis was applied for assessment of the relation between inhibition of JAK activity and cytokine signaling. Data for the correlation analysis, i.e., IC_50_ values of JAK activity and IC50u values of cytokine signaling, was from the present study and from earlier, identical, analyses of inflammatory JAK inhibitors.^24^

### Generation of Humanized JAK2 Ba/F3 Cell Lines

Ba/F3 cell lines were cultured in RPMI 1640 medium (Thermo Fisher Scientific) supplemented with 10% FBS and 10 ng/mL recombinant mouse IL-3 (Peprotech) or 10% conditioned medium from the WEHI-3B cell line. Ba/F3-hJAK2 wild-type and Ba/F3 hJAK2 V617F cells were engineered from the parental Ba/F3 cell line. First, the parental Ba/F3 cell line was transduced with hJAK2 wild-type or hJAK2 V617F cDNA constructs embedded in the retroviral MSCV-IRES-GFP backbone. GFP-positive cells were sorted 48 h after transduction using a BD FACSAria III Cell Sorter (BD Biosciences). After recovery and expansion, the mJak2 locus was disrupted in Ba/F3-hJAK2 wild-type and Ba/F3-hJAK2 V617F cells using CRISPR-Cas9 gene editing. Guide RNA targeting exon 4 of mouse Jak2 (5′-TGTGGAAGACATGATTGGGT-3′) was selected based on its low level of homology to human JAK2 to prevent hJAK2 targeting and was subsequently cloned into the lentiviral backbone pL-CRISPR.EFS.tRFP (Addgene #57819). 2 × 10^6^ sample of humanized Ba/F3 cells were subjected to the electroporation with 15 μg of the vector using Cell Line Nucleofector Kit V (Lonza). Double GFP- and RFP-positive cells were sorted 72 h after nucleofection in bulk. After recovery and expansion, single-cell Ba/F3 clones were sorted into 96-well plates using a BD FACSAria III Cell Sorter (BD Biosciences). Genomic DNA from single-cell Ba/F3 clones was used to confirm the efficient disruption of all three mouse Jak2 alleles. Briefly, the targeted locus was polymerase chain reaction-amplified using Q5 polymerase (NEB) and the following primers: mJAK2 FWD 5′-GTTTTAGGGAGTGTTTTCC-3′ and mJAK2 REV 5′- CTCCTGGGAAACTGGCAATA-3′. Subsequently, the PCR fragments were subcloned by using a PCR cloning kit (NEB) and subjected to Sanger sequencing (Microsynth). Human JAK2 expression was further characterized in selected clones by flow cytometry, Western Blotting using a JAK2 monoclonal antibody (clone 1C1, Thermo Fisher Scientific), and a proliferation assay using CellTiter-Glo (Promega).

### Ba/F3 Viability Inhibition Assay

The effect of JAK inhibitors on viability of Ba/F3-hJAK2 wildtype and V617F cells was studied as described previously.^46^ Reactions were performed in triplicate, and data presented are averages of three individual experiments.

### Structure Determination

JAK2 kinase domain was crystallized as described by Andraos et al.^47^ Briefly, the JAK2–inhibitor complexes were acquired by cocrystallization. The inhibitors (300 μM) were mixed with JAK2 (7–8 mg/mL) and incubated on ice for 30 min before setting up the crystallization drops. The crystallization was done in hanging-drop vapor diffusion method by mixing equal volumes of protein–inhibitor complex with well solution (1.4–2.4 M Na-malonate, pH 6, 0.1 M glycyl–glycine, pH 8.2). Before data collection, the crystals were briefly soaked in a well solution with 2.7 M sodium malonate and flash frozen in liquid nitrogen. Diffraction data were collected on beamline I03 at Diamond Light source, Didcot, UK. Data were processed and scaled with XDS^48^ and Aimless.^49^ Structures were determined by molecular replacement with Phaser^50^ using a 6VN8 as a search model. The structure refinement and model building were done with phenix.refine^51^ and Coot.^52^ Data collection and refinement statistics are listed in Table S5.

### Isothermal Titration Calorimetry

ITC was done with VP-ITC (Malvern Panalytical Ltd., UK) at 21 °C. The titrations were performed in 20 mM *N*-(2-hydroxyethyl)piperazine-*N*′-ethanesulfonic acid pH 8.0, 150 mM NaCl, 10% glycerol, 0.5 mM TCEP with 3–20 μM of protein, and 30–200 μM of the inhibitor. Data were analyzed with Origin 7 (OriginLab).

### Binding Site Water Mapping

Ligand site solvation was determined with WATsite 3.0.^53^ For each JAK2–ligand complex, a separate simulation with an equilibration phase of 1 ns and a production stage of 5 ns at 294.15 K was run. Two fs time steps were used, and frames were collected every 5 ps. A nonbonded interaction cutoff of 10 Å was used, and long-range interactions were treated with the Particle Mesh Ewald method. Heavy atoms were restrained with a spring constant of 2.5 kcal/mol/Å^2^.

## Supporting Information Available

Supplementary methods, tables, figures, and references (PDF)

## Accession Codes

Coordinates and structure factors of protein crystal structures are available at Protein Data Bank (http://www.rcsb.org) with accession codes 8BX6 (JAK2 JH1-cerdulatinib), 8BX9 (JAK2 JH1-ilginatinib), 8BXC (JAK2 JH1-itacitinib), 8BXH (JAK2 JH1-momelotinib), 8BPV (JAK2 JH1-pacritinib), 8BM2 (JAK2 JH1-gandotinib), and 8BPW (JAK2 JH1-lestaurtinib.

## Abbreviations

ACVR1: activin receptor type-1
ALK2: activin receptor-like kinase-2
CALR: calreticulin
ET: essential thrombocythemia
GVHD: graft versus host disease
ITC: isothermal titration calorimetry
JAK: Janus kinase
JAKinib: JAK2 inhibitor
MPL: thrombopoietin receptor
MPN: myeloproliferative neoplasm
PMF: primary myelofibrosis
PV: polycythemia vera
TYK2: tyrosine kinase 2
ULK3: Unc-51-like kinase 3
