# Supplementary material for: Functional and Structural Characterization of Clinical-Stage Janus Kinase 2 Inhibitors Identifies Determinants for Drug Selectivity
Source: J Med Chem. 2024 Jun 6;67(12):10012–24. doi: 10.1021/acs.jmedchem.4c00197 (PMC11215726; doi:10.1021/acs.jmedchem.4c00197)
Supplement: Supplementary file 1 — jm4c00197_si_001.pdf [file jm4c00197_si_001.pdf]

Supporting Information

Functional and Structural Characterization of Clinical-Stage Janus Kinase 2 Inhibitors Identifies Determinants for Drug Selectivity

Ya Miao[1#], Anniina Virtanen[1,2#], Jakub Zmajkovic[3], Morgane Hilpert[3], Radek C. Skoda[3], Olli Silvennoinen[1,2,4]*, Teemu Haikarainen[1,4]*

1Faculty of Medicine and Health Technology, Tampere University, 33520, Tampere, Finland.

2Institute of Biotechnology, HiLIFE, University of Helsinki, 00790, Helsinki, Finland.

3Experimental Hematology, Department of Biomedicine, University Hospital Basel and University of Basel, 4056 Basel, Switzerland

4Fimlab Laboratories, 33520, Tampere, Finland.

[#]These authors contributed equally to this work
*Corresponding authors:
Teemu Haikarainen: teemu.haikarainen@tuni.fi
Olli Silvennoinen: olli.silvennoinen@tuni.fi

Table of Contents

Supplementary Methods S2

Supplementary Tables S3-S8

Supplementary Figures S9-S17

Supplementary References S18

Supplementary Methods

Thermodynamics of JAK2-gandotinib interaction

Due to poor solubility of gandotinib in assay buffer and low enthalpy change of the binding reaction, the binding of gandotinib to JAK2 JH1 was analyzed with fluorescence polarization combined with ITC. Gandotinib binding to JAK2 JH1 was measured with fluorescence polarization (Table 1). The ΔG was calculated from $\Delta G = -RTlnK$ using K derived from fluorescence polarization measurement. Enthalpy change was derived from the incomplete gandotinib ITC data (Figure S7). $-\Delta TS$ was calculated from $\Delta G = \Delta H - \Delta TS$.

Supplementary Tables

Table S1. Binding of JAK inhibitors on pseudokinase domain of JAK family members.

	Binding affinity k_d [nM]			
	JAK1 JH2	JAK2 JH2	JAK3 JH2	TYK2 JH2
ruxolitinib	ND	ND	ND	ND
momelotinib	4990	ND	ND	3048
ilginatinib	2408	ND	ND	5809
itacitinib	14389	ND	ND	ND
cerdulatinib	1.4	994	5105	15
pacritinib	583	ND	ND	29
lestaurtinib	ND	ND	ND	ND
gandotinib	9363	ND	ND	ND
fedratinib	3386	ND	ND	ND
AT9283	38	2870	5670	12

Data is derived from fluorescence polarization binding assay and presented as average of triplicate samples from 3 individual experiments. Numbers are the nanomolar binding affinity (kd) of a JAKinib for JAK family members. ND, inhibition not detected or IC_{50} >20000 nM.

Table S2. Selectivity of inhibitors in inhibition of cytokine signaling.

	TF-1	Human whole blood									
	EPO JAK2	IL-2 JAK1/3		IL-6 JAK1		IFN-α JAK1/TYK2		IFN-γ JAK1/JAK2		GM-CSF JAK2	
	IC_{50}	IC_{50}	IC_{50u}	IC_{50}	IC_{50u}	IC_{50}	IC_{50u}	IC_{50}	IC_{50u}	IC_{50}	IC_{50u}
ruxolitinib	5	383	11	1275	38	171	5.1	1398	42	2561	77
momelotinib	86	411	78	457	87	412	78	379	72	22850	4342
ilginatinib	41	1964	NA	3182	NA	1128	NA	696	NA	6536	NA
itacitinib	488	93	24	315	82	264	69	205	53	4420	1149
cerdulatinib	99	237	52	377	83	127	28	2146	472	21047	4630
pacritinib	352	12572	126	23817	238	4810	48	39727	397	194210	1942
lestaurtinib	26	1171	12	3625	36	2802	28	5835	58	27666	277
gandotinib	155	1281	NA	4715	NA	1175	NA	2744	NA	8842	NA
fedratinib	457	664	60	1008	91	821	74	4212	379	4006	361
AT9283	21	18	NA	104	NA	13	NA	11	NA	316	NA

EPO-pSTAT5 inhibition was measured in TF-1 cells. IFN-α-pSTAT1, IL-6-pSTAT3, IFN-γ-pSTAT1, and GM-CSF-pSTAT5 inhibition was measured in human whole blood CD33+ monocytes, and IL-2-pSTAT5 inhibition in CD4+ T cells. Numbers are the nanomolar half-maximal inhibitory concentration (IC_{50}) or unbound IC_{50} (IC_{50}u) of a JAKinib for cytokine signaling. NA, binding tendency for plasma proteins unknown.

Table S3. Inhibition of inflammatory JAK inhibitors for EPO-pSTAT5 signaling in TF-1 cells in comparison with unbound IC_{50} values for GM-CSF-pSTAT5 and IFN-γ-pSTAT1 in blood.

	EPO IC_{50} [nM]	GM-CSF $IC_{50}u$ [nM]	IFN-γ $IC_{50}u$ [nM]
tofacitinib	92	279	38
peficitinib	126	424	55
filgotinib	1963	4746	1204
upadacitinib	54	244	28
abrocitinib	1077	2531	405
ritlecitinib	ND	ND	ND
decernotinib	645	NA	NA
baricitinib	19	41	9
deucravacitinib	ND	3122	382
brepocitinib	80	336	67

Data is average of three independent experiments. GM-CSF and IFN-γ data from publication.[1] ND, inhibition not detected or IC_{50} > 50 µM. NA, not analysed (unbound fraction data not available). Unbound fractions used in the calculation of $IC_{50}u$ were: 0.61 for tofacitinib,[2] 0.26 for peficitinib,[3] 0.45 for filgotinib,[4] 0.48 for upadacitinib,[5] 0.36 for abrocitinib,[6] 0.86 for ritlecitinib,[7] 0.5 for baricitinib,[8] 0.14 for deucravacitinib,[9] and 0.61 for brepocitinib.[10]

Table S4. Correlation between inhibition of JAK-family members and inhibition of cytokine signaling.

JAK family member	Cytokine signaling					
	EPO		GM-CSF		IFN-γ	
	Pearson coeff.	P-value	Pearson coeff.	P-value	Pearson coeff.	P-value
JAK1	0.2759	0.2837 (ns)	0.3713	0.2116 (ns)	0.3334	0.2656 (ns)
JAK2	0.7322	0.0008 (**)	0.5542	0.0494 (ns)	0.8476	0.0003 (**)
JAK3	0.2422	0.3489 (ns)	-0.03813	0.9063 (ns)	-0.1047	0.7460 (ns)
TYK2	0.3036	0.2362 (ns)	-0.05832	0.8499 (ns)	0.1424	0.6425 (ns)

Pearson coefficient and P-value data from Pearson correlation analysis for JAK activity and cytokine signaling. Data in the analysis: kinase activity IC50 for each JAK family member (Table 1), EPO IC_{50} (Figure 1a, Table S3), GM-CSF IC_{50}u or IFN-γ IC_{50}u (Table S1, Table S3). P-value summary shown in parenthesis: ns, p-value >0.0332, * <0.0332, ** <0.0021, *** <0.0002, **** <0.0001.

Table S5. Data collection and refinement statistics.

	**JAK2-cerdulatinib (PDB code 8BX6)**	**JAK2-ilginatinib (PDB code 8BX9)**	**JAK2-itacitinib (PDB code 8BXC)**	**JAK2-momelotinib (PDB code 8BXH)**
**Data**				
Beam line	Diamond I03	Diamond I03	Diamond I03	Diamond I03
Wavelength (Å)	0.97625	0.97625	0.97625	0.97625
Space group	C2	I2	I2	C2
Cell dimensions				
a, b, c (Å)	107.89, 69.23, 50.27	99.53, 68.88, 107.96	99.44, 68.95, 109.89	107.36, 68.93, 49.94
α, β, γ (°)	90.00, 99.33, 90.00	90.00, 99.13, 90.00	90.00, 99.57, 90.00	90.00, 99.51, 90.00
Resolution (Å)	58-1.50 (1.52-1.50)	78-1.40 (1.42-1.40)	79-1.90 (1.94-1.90)	57-1.30 (1.32-1.30)
$CC_{1/2}$ (%)	99.9 (67.6)	100 (80.9)	99.7 (74.6)	100 (68.2)
$I/\sigma I$	16.2 (1.4)	9.8 (1.3)	9.4 (1.7)	18.9 (1.3)
R_{merge}	0.066 (1.343)	0.075 (1.271)	0.140 (1.172)	0.046 (0.992)
Completeness (%)	99.5 (98.7)	99.7 (97.4)	99.8 (98.8)	99.1 (85.4)
Redundancy	6.9 (6.8)	6.9 (6.9)	6.9 (6.8)	6.7 (4.7)
**Refinement**				
Reflections	58187	140998	57759	87320
R_{work}/R_{free}	0.174/0.202	0.191/0.223	0.184/0.212	0.145/0.168
RMSD of bond lengths (Å)	0.015	0.012	0.005	0.007
RMSD of bond angles (°)	1.460	1.086	0.895	1.118
B-factors ($Å^2$)				
Protein	27.49	21.89	28.33	24.27
Ligand	44.9	16.65	28.65	33.0

Values for the highest-resolution shell are shown in parentheses.

Table S5. Data collection and refinement statistics. Continued

	**JAK2-pacritinib (PDB code 8BPV)**	**JAK2- gandotinib (PDB code 8BM2)**	**JAK2-lestaurtinib (PDB code 8BPW)**
**Data**			
Beam line	Diamond I03	Diamond I03	Diamond I03
Wavelength (Å)	0.97625	0.97625	0.97625
Space group	C2	I2	I2
Cell dimensions			
a, b, c (Å)	108.07, 68.96, 49.96	99.89, 69.25, 110.30	99.53, 69.51, 110.89
α, β, γ (°)	90.00, 98.85, 90.00	90.00, 98.68, 90.00	90.00, 98.70, 90.00
Resolution (Å)	57–1.70 (1.73–1.70)	58–1.50 (1.53–1.50)	68–1.80 (1.84–1.80)
$CC_{1/2}$ (%)	99.9 (69.3)	99.9 (50.3)	99.9 (76.2)
$I/\sigma I$	13.7 (1.8)	12.7 (1.0)	14.7 (1.9)
R_{merge}	0.086 (1.115)	0.092 (1.775)	0.096 (1.014)
Completeness (%)	(100) 99.96	(100) 99.91	(100) 99.83
Redundancy	6.9 (7.1)	6.9 (6.7)	6.9 (7.1)
**Refinement**			
Reflections	39953	118905	69405
R_{work}/R_{free}	0.172/0.198	0.177/0.202	0.161/0.197
RMSD of bond lengths (Å)	0.012	0.016	0.011
RMSD of bond angles (°)	1.056	1.323	1.222
B-factors			
Protein	32.49	25.79	29.08
Ligand	38.60	28.90	21.90

Values for the highest-resolution shell are shown in parentheses.

Supplementary Figures

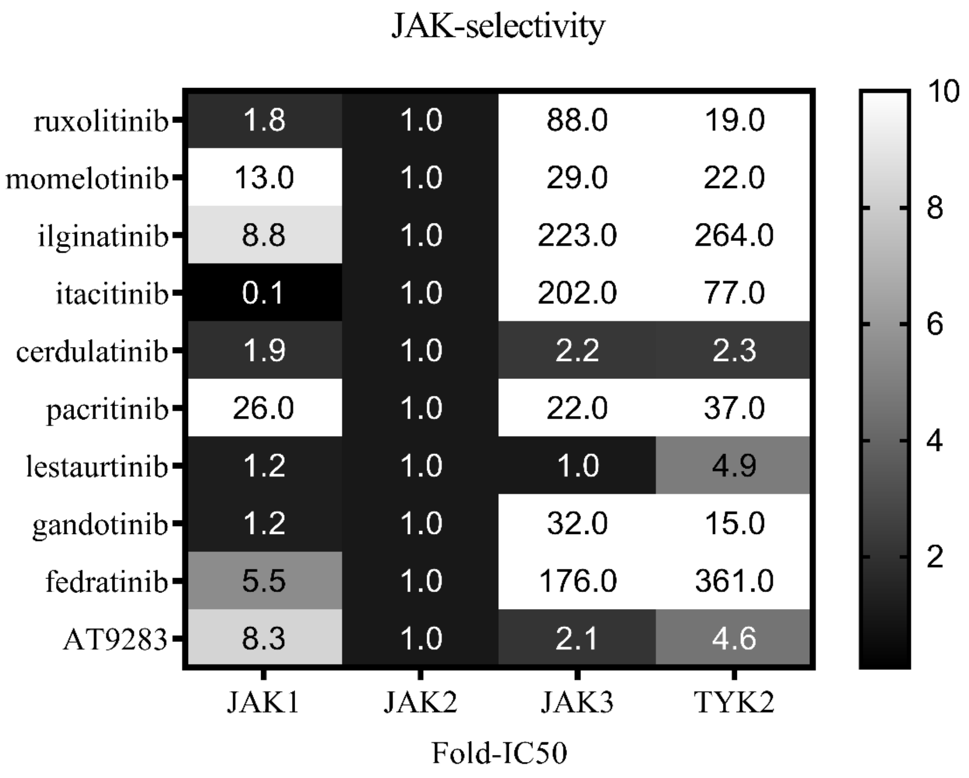

Figure S1. JAK-selectivity of JAK inhibitors. Data presented is mean fold IC_{50} for catalytic activity of each JAK family member normalized to inhibition of JAK2 activity.

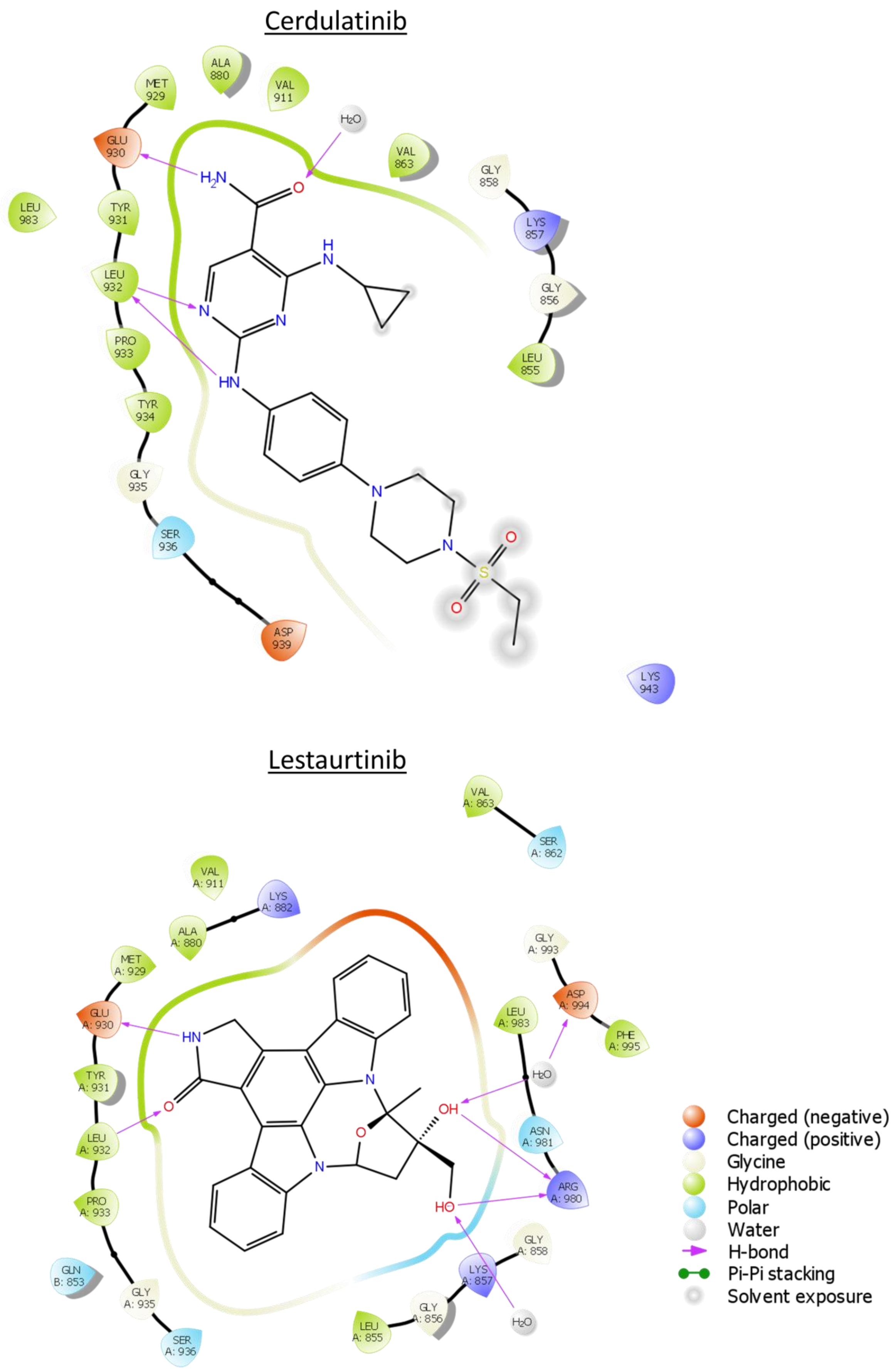

Figure S2. Structures and binding environments of cerdulatinib and lestaurtinib.

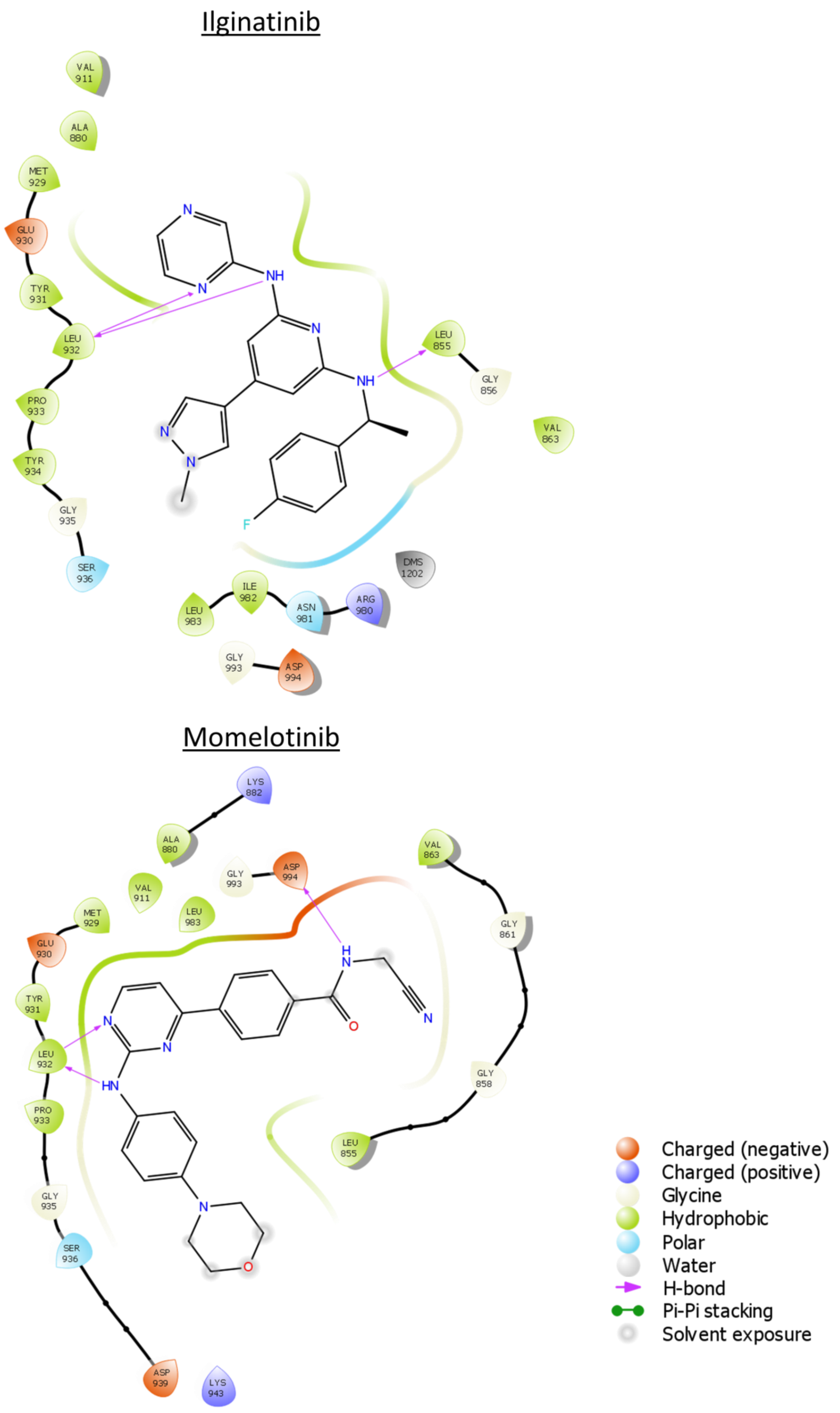

Figure S3. Structures and binding environments of ilginatinib and momelotinib.

Gandotinib

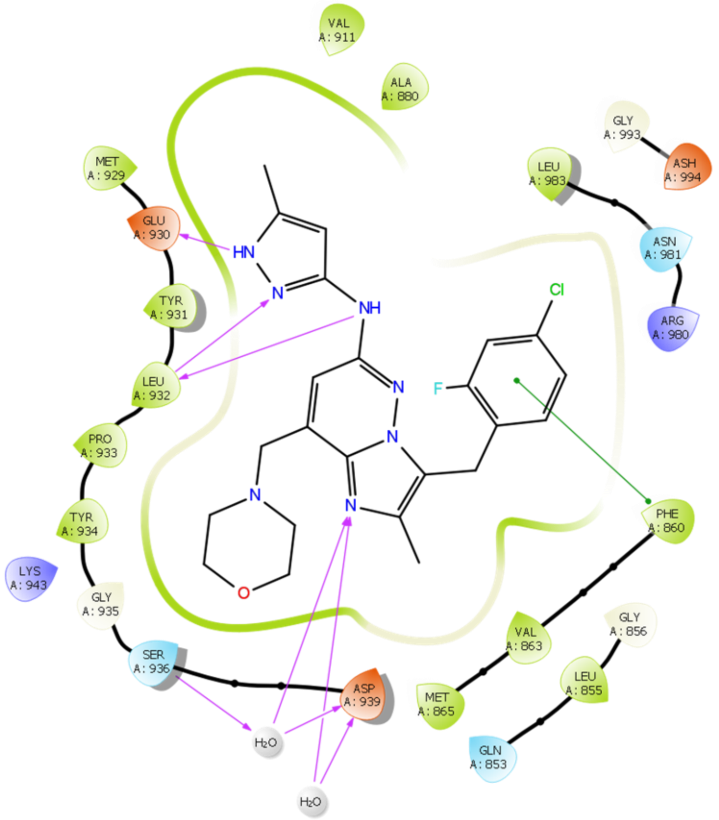

Pacritinib

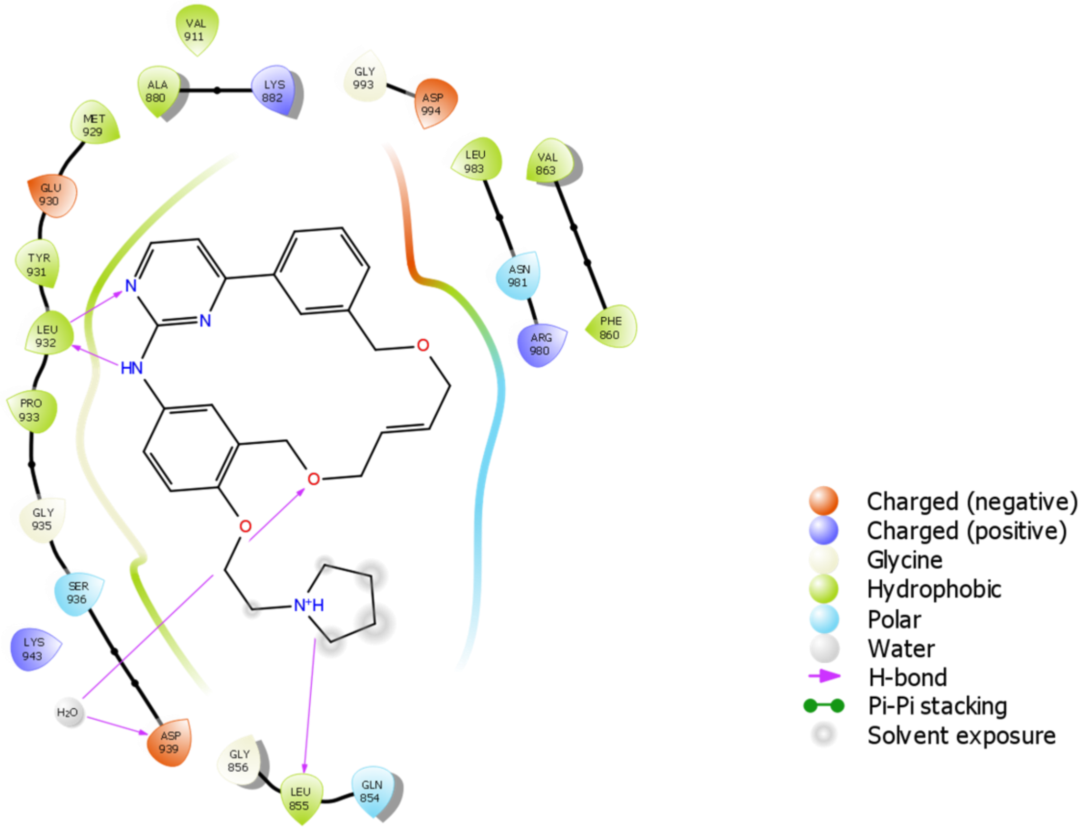

Figure S4. Structures and binding environments of gandotinib and pacritinib.

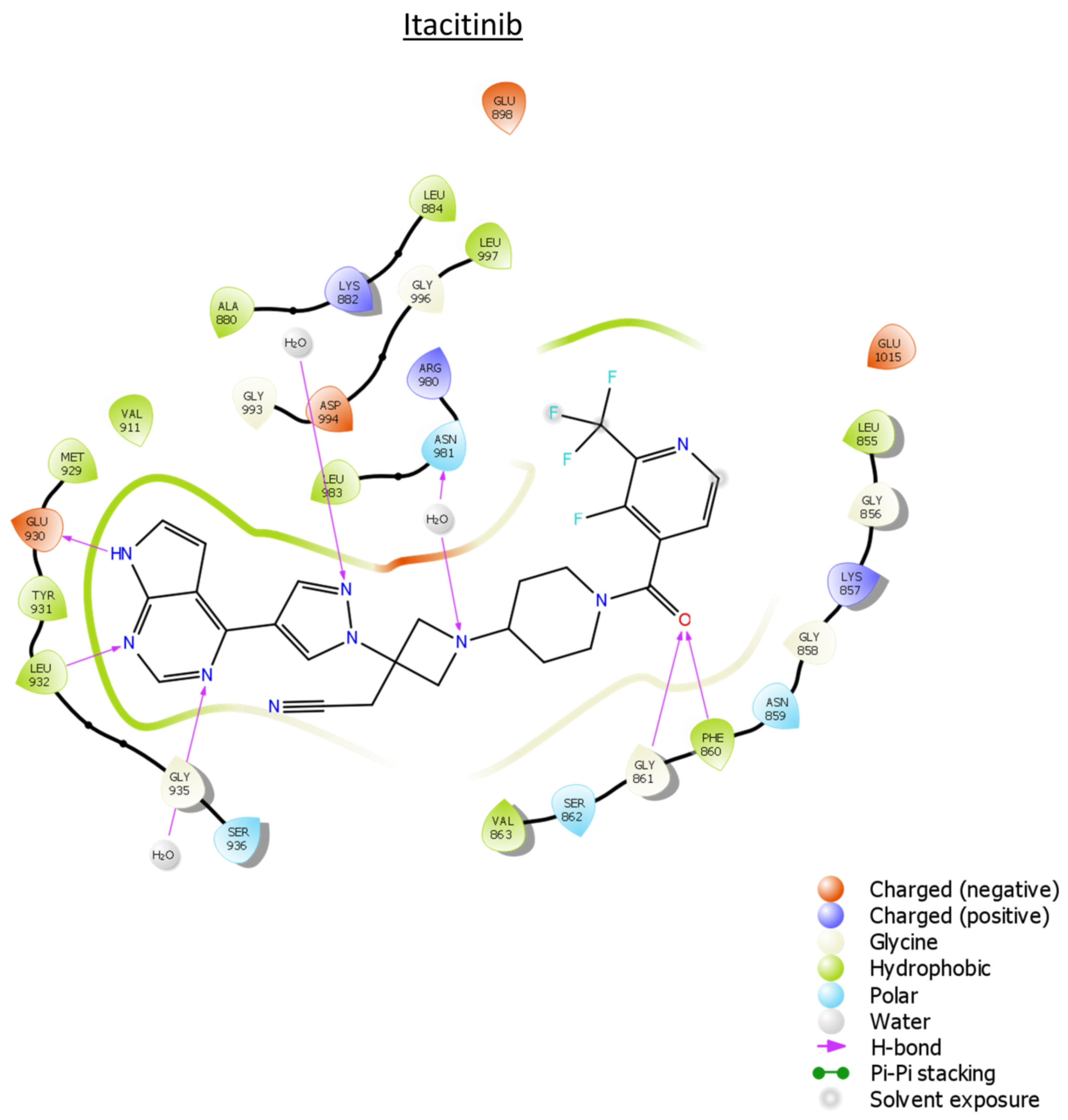

Figure S5. Structure and binding environment of itacitinib.

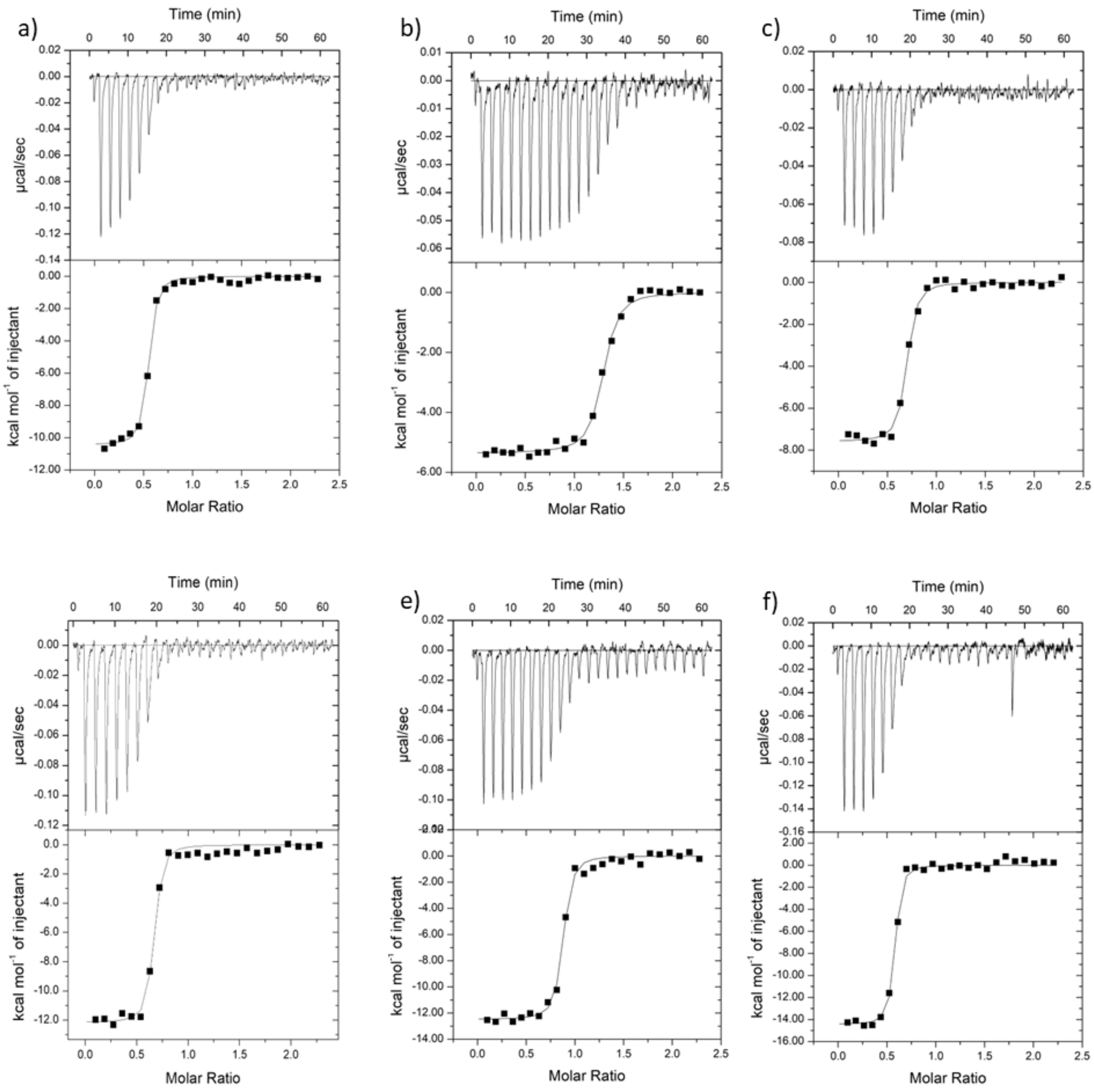

Figure S6. ITC measurements. a) cerdulatinib, b) itacitinib, c) pacritinib, d) lestaurtinib, e) ilginatinib, f) momelotinib.

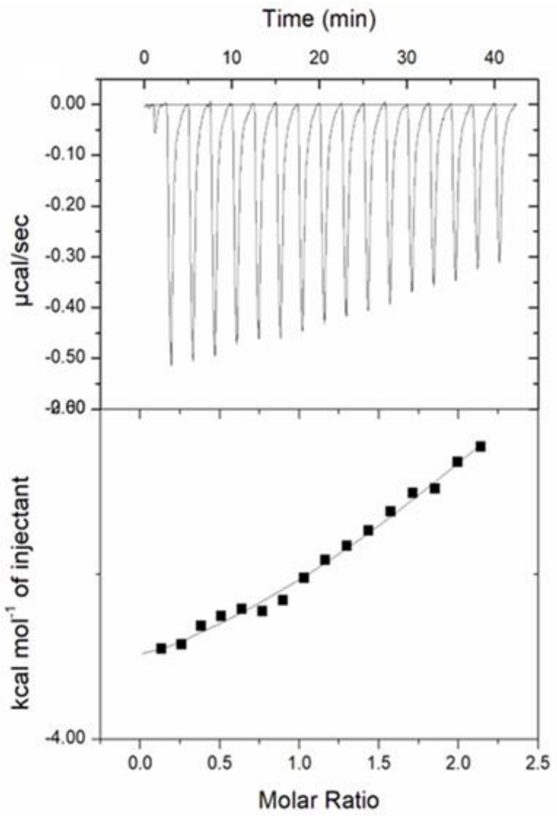

Figure S7. ITC analysis of JAK2-gandotinib interaction.

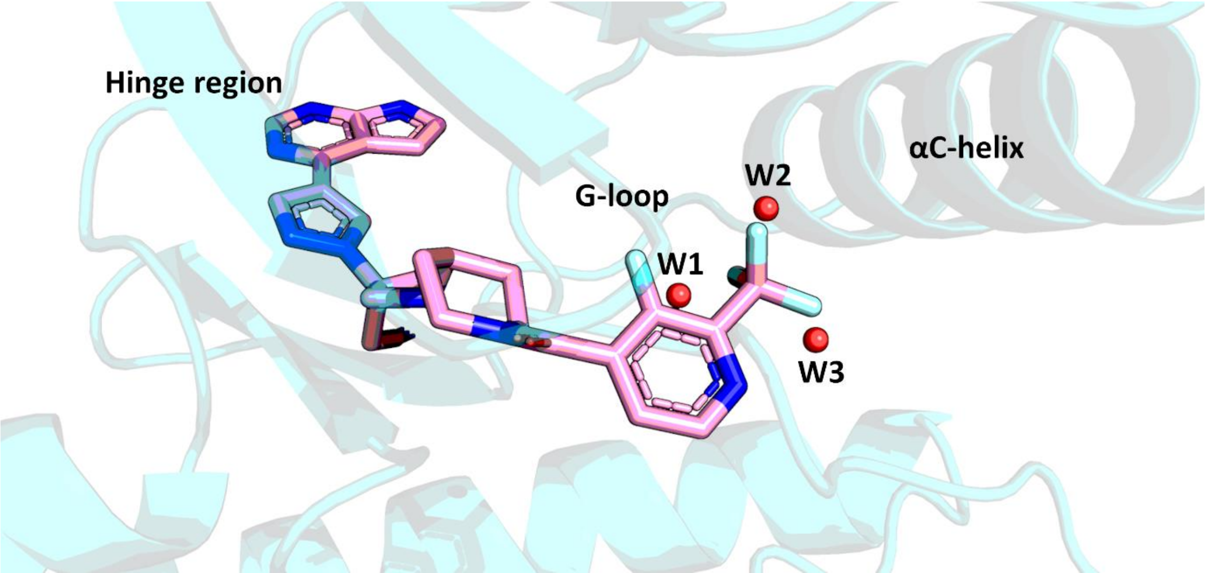

Figure S8. Water molecules displaced by itacitinib at the tip of the G-loop. JAK2-itacitinib structure showing superposed water molecules (W1-W3) from JAK2-momelotinib complex. The binding of itacitinib leads to displacement of these water molecules.

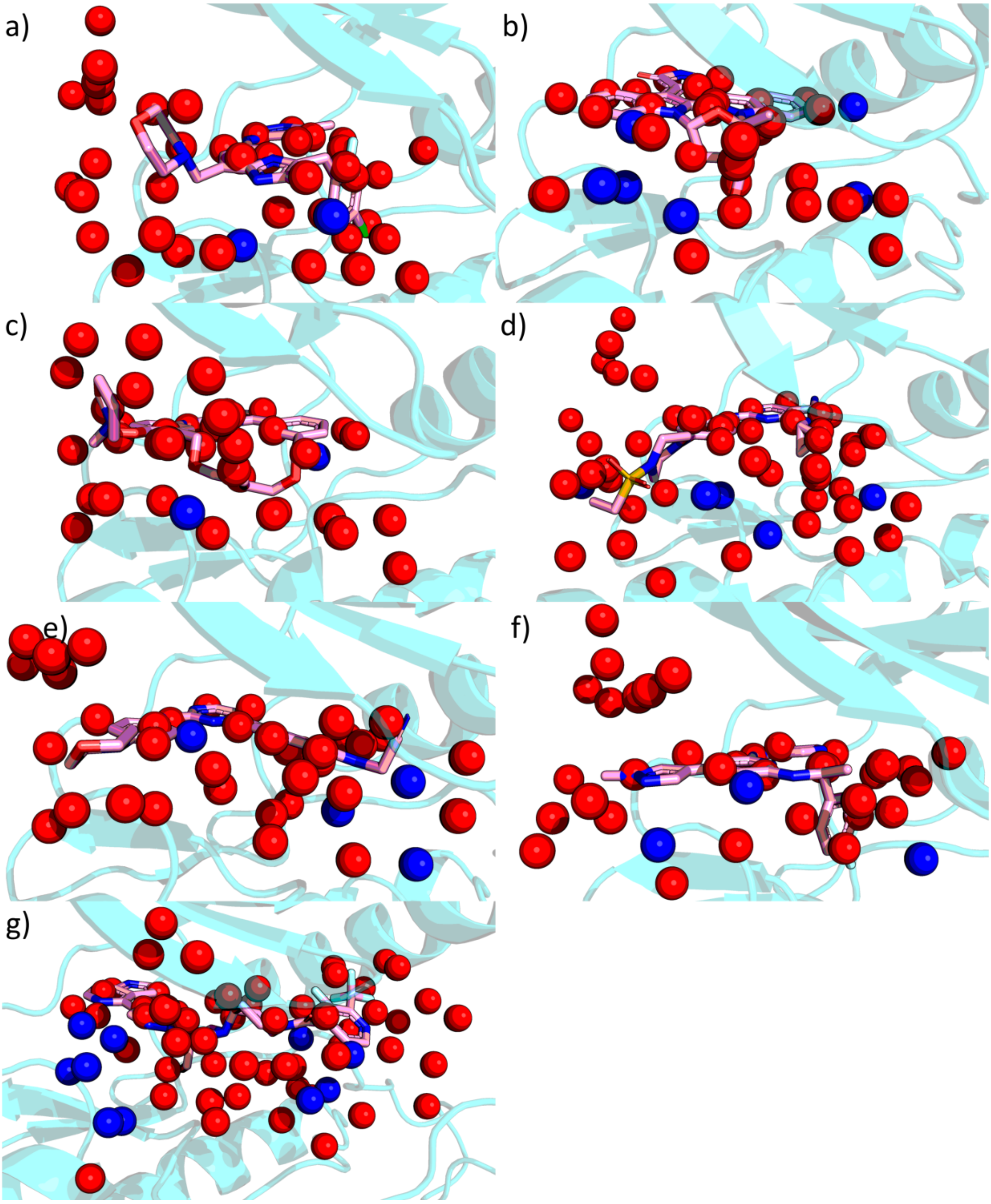

Figure S9. Binding site water analysis with WATsite. a) cerdulatinib, b) itacitinib, c) ilginatinib, d) lestaurtinib, e) momelotinib, f) gandotinib, and g) pacritinib. JAK2 is shown in cyan and ligand in pink. Waters occupying the inhibitor binding pocket are shown in red (unfavorable, ΔG > 0) and blue (favorable, ΔG < 0).

Supplementary References

(1) Virtanen, A.; Palmroth, M.; Liukkonen, S.; Kurttila, A.; Haikarainen, T.; Isomäki, P.; Silvennoinen, O. Differences in JAK Isoform Selectivity Among Different Types of JAK Inhibitors Evaluated for Rheumatic Diseases Through In Vitro Profiling. *Arthritis Rheumatol* **2023**, *75* (11), 2054–2061. https://doi.org/10.1002/art.42547.
(2) Pfizer Inc. Prescribing Information XELJANZ (Tofacitinib), 2018. https://www.accessdata.fda.gov/drugsatfda_docs/label/2018/203214s018lbl.pdf (accessed 2023-04-24).
(3) Pharmaceutical and Medical Devices Agency. Report on the Deliberation Results Smyraf Tablets 50 Mg, Smyraf Tablets 100 Mg, 2019. https://www.pmda.go.jp/drugs/2019/P20190419003/800126000_23100AMX00285_A100_1.pdf (accessed 2023-02-28).
(4) EMA. Summary of Product Characteristics of Jyseleca, 2020. https://www.ema.europa.eu/documents/product-information/jyseleca-epar-product-information_en.pdf (accessed 2023-04-24).
(5) EMA. Assessment Report Rinvoq, 2020. https://www.ema.europa.eu/en/documents/assessment-report/rinvoq-epar-public-assessment-report_en.pdf (accessed 2023-02-28).
(6) Bauman, J. N.; Doran, A. C.; King-Ahmad, A.; Sharma, R.; Walker, G. S.; Lin, J.; Lin, T. H.; Telliez, J.-B.; Tripathy, S.; Goosen, T. C.; Banfield, C.; Malhotra, B. K.; Dowty, M. E. The Pharmacokinetics, Metabolism, and Clearance Mechanisms of Abrocitinib, a Selective Janus Kinase Inhibitor, in Humans. *Drug Metab Dispos* **2022**, *50* (8), 1106–1118. https://doi.org/10.1124/dmd.122.000829.
(7) Robinson, M. F.; Damjanov, N.; Stamenkovic, B.; Radunovic, G.; Kivitz, A.; Cox, L.; Manukyan, Z.; Banfield, C.; Saunders, M.; Chandra, D.; Vincent, M. S.; Mancuso, J.; Peeva, E.; Beebe, J. S. Efficacy and Safety of PF-06651600 (Ritlecitinib), a Novel JAK3/TEC Inhibitor, in Patients With Moderate-to-Severe Rheumatoid Arthritis and an Inadequate Response to Methotrexate. *Arthritis Rheumatol* **2020**, *72* (10), 1621–1631. https://doi.org/10.1002/art.41316.
(8) EMA. Assessment Report Olumiant, 2017. https://www.ema.europa.eu/documents/assessment-report/olumiant-epar-public-assessment-report_en.pdf (accessed 2023-11-04).
(9) Bristol-Myers Squibb Company. Prescribing Information Sotyktu (Deucravacitinib,TYK2i), 2022. https://packageinserts.bms.com/pi/pi_sotyktu.pdf (accessed 2023-02-28).
(10) Banfield, C.; Scaramozza, M.; Zhang, W.; Kieras, E.; Page, K. M.; Fensome, A.; Vincent, M.; Dowty, M. E.; Goteti, K.; Winkle, P. J.; Peeva, E. The Safety, Tolerability, Pharmacokinetics, and Pharmacodynamics of a TYK2/JAK1 Inhibitor (PF-06700841) in Healthy Subjects and Patients With Plaque Psoriasis. *The Journal of Clinical Pharmacology* **2018**, *58* (4), 434–447. https://doi.org/10.1002/jcph.1046.
